# Comparative analysis of weighted gene co-expression networks in human and mouse

**DOI:** 10.1371/journal.pone.0187611

**Published:** 2017-11-21

**Authors:** Marius Eidsaa, Lisa Stubbs, Eivind Almaas

**Affiliations:** 1 Department of Biotechnology, NTNU - Norwegian University of Science and Technology, N-7491 Trondheim, Norway; 2 Institute for Genomic Biology, Neuroscience Program, Cell and Developmental Biology, University of Illinois at Urbana-Champaigne, Urbana, IL 61801, United States of America; 3 K.G. Jebsen Center for Genetic Epidemiology, Department of Public Health and General Practice, NTNU - Norwegian University of Science and Technology, Trondheim, Norway; University of Texas at San Antonio, UNITED STATES

## Abstract

The application of complex network modeling to analyze large co-expression data sets has gained traction during the last decade. In particular, the use of the weighted gene co-expression network analysis framework has allowed an unbiased and systems-level investigation of genotype-phenotype relationships in a wide range of systems. Since mouse is an important model organism for biomedical research on human disease, it is of great interest to identify similarities and differences in the functional roles of human and mouse orthologous genes. Here, we develop a novel network comparison approach which we demonstrate by comparing two gene-expression data sets from a large number of human and mouse tissues. The method uses weighted topological overlap alongside the recently developed network-decomposition method of *s*-core analysis, which is suitable for making gene-centrality rankings for weighted networks. The aim is to identify globally central genes separately in the human and mouse networks. By comparing the ranked gene lists, we identify genes that display conserved or diverged centrality-characteristics across the networks. This framework only assumes a single threshold value that is chosen from a statistical analysis, and it may be applied to arbitrary network structures and edge-weight distributions, also outside the context of biology. When conducting the comparative network analysis, both within and across the two species, we find a clear pattern of enrichment of transcription factors, for the homeobox domain in particular, among the globally central genes. We also perform gene-ontology term enrichment analysis and look at disease-related genes for the separate networks as well as the network comparisons. We find that gene ontology terms related to regulation and development are generally enriched across the networks. In particular, the genes FOXE3, RHO, RUNX2, ALX3 and RARA, which are disease genes in either human or mouse, are on the top-10 list of globally central genes in the human and mouse networks.

## Introduction

Mouse is the dominant model organism in biomedical research focused on understanding human disease. Despite this fact, humans and mice differ both in genome content and organization as well as gene expression profiles [1, 2]. Understanding similarities and differences in system-level organization, and in particular regulatory mechanisms, for human and mouse orthologs is therefore of great importance. In particular, the mammalian central nervous system (CNS), which includes the brain and spinal cord, shows great species variation in terms of cell types, physiology and function [3]. Knowledge about the similarities and differences in functional organization of human and mouse brains, is thus important for interpreting the results from mouse models for human neurodevelopment, cognitive function and behavior [4].

Gene co-expression network analysis has recently been successfully applied to a wide range of biological systems, providing insight into the regulatory nature of genes and gene products (see e.g. [5–11]). The standard approach of gene co-expression network analysis, more well known as weighted gene co-expression network analysis (WGCNA) [12–14], is most often based on the three following steps [14]: (1) the application of a similarity measure (e.g. a correlation measure, calculation of mutual information, or regression analysis) for each pairing of genes to develop a similarity matrix, (2) implementation of a threshold value on the similarity scores so that the resulting network exhibits scale-free topology and finally (3) performing hierarchical clustering, e.g. with respect to functional organization. The resulting networks are utilized to uncover information about potential regulatory pathways and functional gene clusters. Consequently, the network role of transcription factors (TFs) and their interaction partners are of particular interest because of their inherent importance in gene regulation.

The availability of large-scale gene-expression data sets in human and mouse has made system-level studies of co-expression networks a promising avenue of investigation. Several recent studies have compared human and mouse co-expression networks using a wide range of network approaches [15–18]. Their results suggest that both tissue type and gene function deeply affect evolutionary conserved gene clusters, predictability of disease-relevant relationships between human and mouse, preservation of developmental stage-specific modules in human and mouse embryos, and several conserved and diverging gene-expression network properties.

Although the weighted gene co-expression network analysis (WGCNA) [12–14] is an established and well-tested analysis framework that has provided several meaningful biological insights, it is not without limitations. Applying correlation thresholds might skew the edge-weights so that only the very strongest correlations are influential, which may not be beneficial in gaining a wide systems understanding of the gene co-expression network. Even though various biological networks display scale-free properties, that does not necessarily imply that inference networks, such as gene co-expression networks, need to be scale free in order to enabling us gaining knowledge about the biological systems in question.

In this study, we analyze human and a mouse microarray data sets, consisting of normalized gene-expression data for multiple tissue types, using an approach initially similar to WCGNA [12–14] by generating four different networks: (1) Human network based on all available tissue types, (2) mouse network based on all available tissue types, (3) human network based on tissue types from CNS tissues and (4) mouse network based on CNS. In contrast to traditional WGCNA, we employ the recently developed *s*-core network peeling approach [19–21] to identify ranked sets of genes of central importance in the networks. As a weighted generalization of the *k*-core network decomposition method [22], the *s*-core decomposition method works in a similar manner to that of *k*-core, by peeling off the outermost, non-central genes leaving central, highly connected sub-networks called cores. The *s*-core gene-ranking approach mainly differs from the WGCNA counterpart in that it provides a global ranking of genes (i.e. a single ranked list), while WGCNA ranks genes by hierarchical clustering. Throughout the analyses, we aim to keep the number of free parameters and restrictions to a minimum, making the method as data agnostic as possible. We first analyze the four networks individually before conducting a comparative network analysis, utilizing novel network comparison metrics, both within and across the two species. In agreement with previous studies that have shown that most TFs are evolutionarily stable [23], we find enrichment of TFs among globally central genes [24]. We also find strong patterns of enrichment in TF families such as the homeobox protein-coding genes, which have been shown to be highly conserved within human and mouse [25].

## Results

We generated four gene-correlation networks from sets of gene-expression data in multiple tissue types for human and mouse [26] using a weighted topological overlap (wTO) approach (see Methods for details). After removing gene-pair links with weights that are not statistically significant, we were left with gene co-expression networks that are extremely dense (〈*k*〉 ∼ 0.3 ⋅ *N*). In general, this is an often encountered challenge in gene co-expression analyses. One suggestion to solve this problem, is to remove links until the remaining network is scale-free, and thus a very sparse network [14]. However, with such an approach, we get little insight into how the sum of many, relatively weak links may influence important network characteristics, e.g. its community structure.

In contrast, we chose to use the generalization to weighted networks of the *k*-core method for network peeling, *s*-core peeling [19–21], which is based on the sequential removal of the weakest nodes. This approach allows the identification of the most centrally connected regions of a dense network. Thus, we generated four networks that were decomposed into indexed cores using the *s*-core+ method [20], obtaining a ranked node list with the *s*-core index *n* as centrality measure.

### Decomposition analysis of the weighted networks

In the following section, we will first discuss the properties of the four networks [*H*_*A*_ (human all tissues), *H*_*B*_ (human only CNS, abbreviated “brain” below), *M*_*A*_ (mouse all tissues), *M*_*B*_ (mouse only brain)] separately, before we discuss the within-species comparisons of *H*_*A*_ vs. *H*_*B*_ and *M*_*A*_ vs. *M*_*B*_, and the across-species comparisons (*H*_*A*_ vs. *M*_*A*_ and *H*_*B*_ vs. *M*_*B*_). Finally, we will discuss the positioning of disease-associated genes in these networks and network comparisons. Note that the *H*_*B*_ and *M*_*B*_ networks consists solely of TF nodes: Since the mouse brain data only consisted of 10 samples, we focused on the (smaller) gene set of TFs to ensure statistically significant links (see Methods).

Here we should note that both human and mouse data sets are dominated by CNS-related tissues; for example, 21 of the 73 human tissues represented in the arrays correspond to separately analyzed, dissected brain regions [26]. Therefore, we expect the “All-tissues” networks to be strongly influenced by gene relationships found in neuronal tissues. Nevertheless, these networks are also tempered by gene interactions found more widely in the two species. The brain networks, in contrast, should distill out the functions operating more specifically in CNS tissues, and especially in brain.

#### Human all-tissues network

The *H*_*A*_ network consists of all the *N* = 11,896 genes contained in the unperturbed correlation matrix, while the average degree has dropped from 〈*k*〉 = 11,895 (in the fully connected correlation network) to 〈*k*〉 = 7,413 (in the wTO network). The innermost *s*-core (before application of *s*-core+ [20]) contains 5,846 genes, which is about half of the genes of the initial network. Such networks are challenging to analyze and visualize as they are large, dense and highly clustered; by applying *s*-core+, we untangle the network by recursive removal of the smallest link-weights (see “*s*-core+ network decomposition method”). The *s*-core+ network decomposition results in 8,986 distinct *s*-cores, providing the node-centrality sequence *H*_*A*_, where the innermost ten genes (the genes with the highest associated *s*-core index) are shown in Table 1. The *s*-core+ decomposition results follow the node ranking obtained from Eigenvector centrality (EVC) analysis [27, 28] closely, with an overall rank correlation of *ρ* = 0.98. However, among the 1000 innermost genes, there is an overlap of 0.86 between the two methods, signifying increasing disparity between *s*-core+ and EVC as we are getting closer to the innermost part of the network. A scatter plot showing this trend is shown in S1 Fig.

**Table 1:** 

*H*_*A*_	*M*_*A*_	*H*_*B*_	*M*_*B*_
HPSE2	ZSCAN10	DLX4	FOXN1
RHO	PAX9	ZNF669	FEV
NOX1	UBL4B	NR1I2	POU2F3
HTR4	IFNA9	ARNT	ALX4
FAM55D	4931428L18RIK	NKX3-1	FOXE3
MC2R	ZFP628	TP63	SIM2
GML	PAG1	ALX3	ZFP41
RUNX2	WFIKKN1	IRF4	ZFP40
MTMR8	IL2	SPIB	HAND1
OPRM1	FOXE3	TFAP2A	ZFP36L2

The 10 innermost genes in the *s*-core+ decomposition for (left to right) the human all-tissues, mouse all-tissues, human brain, and mouse brain networks presented in ascending order. For an extensive listing, see S1 Table.

#### Mouse all-tissues network

All the original *N* = 15,720 genes are conserved for the *M*_*A*_ network after applying the wTO cutoff (see Methods). With an average degree of 〈*k*〉 = 6,579, the network is slightly sparser than the human network, but still extremely dense. The innermost *s*-core consist of 5,352 genes, but after *s*-core+ decomposition, we obtain a ranked list over all genes, segmented into 9,474 distinct cores. Table 1 displays the 10 innermost genes. The EVC comparison shows the same trend as for human, albeit more pronounced, with a rank correlation of *ρ* = 0.96 of and an overlap of 0.42 among the 1000 innermost genes (see also S2 Fig).

#### Human brain network

The *H*_*B*_ network, constructed from 21 different tissue types from CNS, consists of *N* = 858 nodes, all TFs (see Methods). Thus, some of the original 931 nodes were removed after the wTO link-weight cutoff. The average degree is large, with 〈*k*〉 = 253, so the network is still dense even though some of nodes with small node strengths have been lost. There are only TFs in the brain network due to statistical limitations (see “Network randomization provide statistically significant cutoff values”), but the information from connections between TFs and other genes is kept implicitly due to the wTO approach. There are 306 genes in the innermost *s*-core, and 732 distinct cores after *s*-core+. The top-10 innermost genes in the *H*_*B*_-sequence are presented in Table 1. *s*-core+ gives almost the exact same ranking as EVC for this network, with *ρ* > 0.99 and an overlap of 0.86 among the 100 innermost genes (see S3 Fig).

#### Mouse brain network

Only 10 CNS tissues were available in the construction of the *M*_*B*_ network, resulting in a network with 1071 TF genes and average degree 〈*k*〉 = 130, which is considerably smaller than 〈*k*〉 in the *H*_*B*_ network. The number of nodes in the innermost *s*-core testifies to this, with 167 nodes in the innermost *s*-core. The top-10 most central genes, according to the *s*-core+ decomposition sequence for *M*_*B*_, are shown in Table 1. *s*-core+ and EVC provide similar ranking for this network as well, although with a slightly lower correlation value than the human counterpart, with *ρ* > 0.97. The overlap among the innermost 100 genes, is quite a bit smaller, on the other hand, with 0.46 (see also S4 Fig).

### Transcription factor and gene ontology term enrichment in wTO networks

When conducting enrichment analyses connected to the *s*-core network decomposition, it is important to ensure that the network-peeling process does not have the unintended consequence of removing clusters of genes with high biological significance early in the process. We investigated this possibility by checking for enrichment of GO terms in the outer-most layers of genes in the *s*-core+ sequences for *H*_*A*_, *H*_*B*_, *M*_*A*_ and *M*_*B*_, finding no statistically significant enrichment. This is an indication that the network decomposition process is initially removing biologically unrelated genes.

#### Human all-tissues network

**Fig 1:**
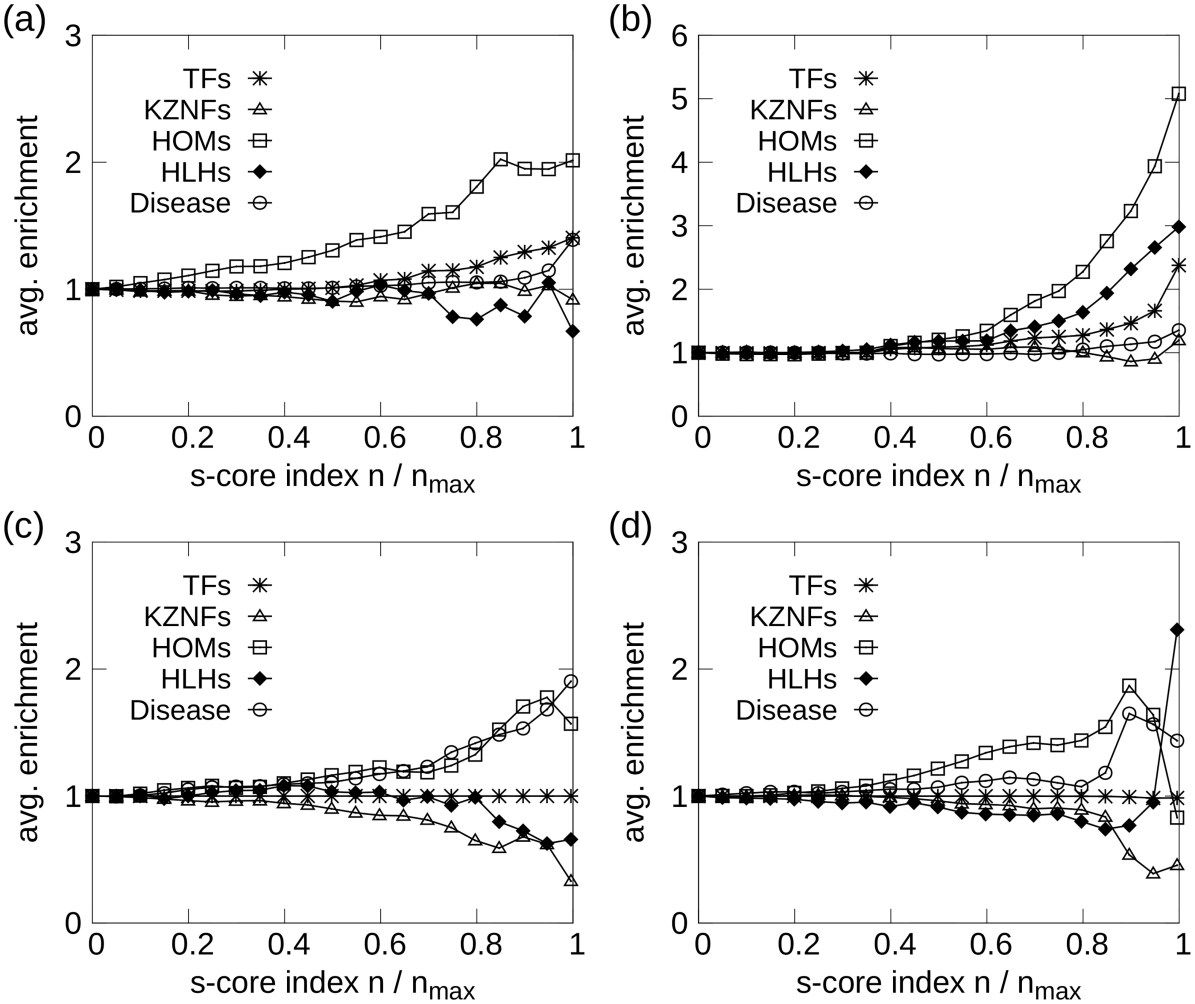
Average enrichment of TFs and selected TF undergroups (see “Comparative enrichment and gene-ontology analysis”) as function of *s*-core index *n* for: (a) Human all-tissues network *H*_*A*_, (b) mouse all-tissues network *M*_*A*_, (c) human brain network *H*_*B*_, and (d) mouse brain network *M*_*B*_. Large *s*-core index values indicate high centrality in the network. TFs and HOMs are significantly enriched for both human and mouse all-tissues networks. The enrichment of TFs is trivially 1 for the brain networks.

Fig 1(a) shows the enrichment of transcription factors and selected TF families for the *H*_*A*_ network, as function of the normalized *s*-core index. The panels shows that TFs are central in the network, with steadily increasing enrichment from *n*/*n*_max_ ∼ 0.5, reaching a 1.5-fold enrichment for the 500 innermost genes. The subfamily of transcription factor genes, homeobox (HOM), are strongly enriched in the network, reaching a plateau of 2-fold enrichment for *n*/*n*_max_ > 0.85.

There are multiple, highly enriched GO terms (obtained from GOrilla [29–31]) in the central-most parts of the network: According to the *s*-core+ sequence for *H*_*A*_, we uncovered several enriched GO terms among the 1,000 innermost genes. The enriched process-related terms with Benjamini-Hochberg corrected *p*-values (only those with *p* < 10^−5^) are shown in Table 2. There are several highly enriched GO process terms related to ion- and transmembrane transport, signaling and multicellular organismal processes. For the GO terms related to biological functions, an extensive list is shown in S2 Table, with terms related to molecular transducer activity and transporter activity dominating the list, many with *p* < 10^−8^. It should be noted that the genes related to olfactory function are removed from the GO analysis for the human and mouse all-tissues networks, that is 50 OR-genes for human and 36 OLFR-genes for mouse, neither of which are TFs. These genes share large sequence similarities, likely causing cross-hybridization, which alongside almost identical GO-terms makes them artificially enriched in these particular GO analyses due to clustering in the networks.

**Table 2:** 

GO Term	Description	FDR *p*-value	Enrichment
GO:0032501	multicellular organismal process	2.65E-10	1.47
GO:0055085	transmembrane transport	1.13E-7	1.80
GO:0007186	G-protein coupled receptor signaling pathway	8.14E-7	1.97
GO:0006811	ion transport	1.55E-6	1.68
GO:0007267	cell-cell signaling	1.87E-6	1.98
GO:0044700	single organism signaling	3.12E-6	1.90
GO:0023052	signaling	3.51E-6	1.89
GO:0034220	ion transmembrane transport	5.05E-6	1.83
GO:0099537	trans-synaptic signaling	5.28E-6	2.37
GO:0007600	sensory perception	5.38E-6	2.20
GO:0007268	chemical synaptic transmission	5.60E-6	2.38
GO:0099536	synaptic signaling	5.81E-6	2.37
GO:0098916	anterograde trans-synaptic signaling	6.07E-6	2.38

Enriched GO process terms among the 1,000 central-most genes in the human all-tissues network. *p* < 10^−5^ (Benjamini-Hochberg corrected).

#### Mouse all-tissues network

Fig 1(b) shows substantial enrichment of TFs, and particularly the TF subfamilies of HOM and helix-loop helix (HLH). For *n*/*n*_max_ > 0.5 HLHs and HOMs are greatly enriched, with fold changes > 2 for *n*/*n*_max_ = 0.8. This is in clear contrast to the human networks, where we instead find that the HLHs are weakly suppressed.

We find multiple enriched GO process terms related to multicellular organismal processes, and sensory perception in particular, among the innermost *s*-cores in *M*_*A*_. The process terms with *p* < 10^−3^ are shown in Table 3. Details about the enriched GO function terms can be found in S3 Table, where terms related to molecular transducer activity, and G-protein coupled receptor activity in particular, sequence-specific DNA binding and transcription factor activity top the list.

**Table 3:** 

GO Term	Description	FDR *p*-value	Enrichment
GO:0050953	sensory perception of light stimulus	4.56E-10	4.67
GO:0007601	visual perception	6.31E-10	4.65
GO:0007600	sensory perception	7.37E-10	2.78
GO:0050877	neurological system process	7.76E-6	1.98
GO:0007186	G-protein coupled receptor signaling pathway	2.18E-5	2.02
GO:0009583	detection of light stimulus	2.48E-5	6.40
GO:0032501	multicellular organismal process	1.50E-4	1.35
GO:0007606	sensory perception of chemical stimulus	2.72E-4	4.17
GO:0030901	midbrain development	7.34E-4	5.64
GO:0034587	piRNA metabolic process	7.82E-4	9.14

Enriched GO process terms among the 1,000 centralmost genes in the mouse all-tissues network. *p* < 10^−3^ (Benjamini-Hochberg corrected).

Several of the significant terms in the mouse network are also present for the human all-tissues network, indicating evolutionary conservation of tissue-wide expression of the genes regulating these processes and functions. It should also be noted that we have confirmed that the enrichment profiles are robust with respect to node subset size for both the *H*_*A*_ and the *M*_*A*_ networks.

#### Human brain network

In Fig 1(c), we observe a substantial enrichment of HOMs, which is consistent with our findings for the *H*_*A*_ network. The TF enrichment is trivially 1 for all *n* since all the genes were selected to be TFs. Due to the limited network size, the GO enrichment results are less likely to be deemed statistically significant, but several GO terms, including anion and lipid binding, occur among the 200 innermost genes (see S4 Table).

#### Mouse brain network

In the *M*_*B*_ network (Fig 1(d)) we find enrichment of HOMs until we reach the most central nodes at *n*/*n*_max_ > 0.9. Among the 5 innermost nodes, we find the two HLH genes, HAND1 and SIM2, causing the enrichment increase in HLHs for large *n*-values. HOM genes are highly enriched from *n* ∼ 250 to *n* = 634, where six HOM genes disappear in a cascade of 7 genes. Only a single enriched GO term was found: multicellular organismal development.

### Comparisons of the networks

We have now reported results for the human and mouse network (all tissues or brain) separately, observing similar behavior in some areas, such as enrichment of Homeobox TF genes, while differences in other areas. Here, we will conduct a systematic comparison between the ranked *s*-core+ decomposition sequences for the four wTO networks, using the comparison measures defined in the “Methods” section. Within a species, i.e. when comparing the all-tissues versus the brain networks, the only limiting factor for comparison is the overlapping node content. Since the brain networks only contain TFs, the *s*-core+ sequence-comparisons within a species is limited to TFs. Cross-species comparisons is performed using ortholog data to identify genes to compare.

In order to quantify the difference and similarities in the pair-wise comparison of networks that potentially have the same node content, we focus on using the following measures (see “Methods” for details): The Jaccard index *J*(*S*_1_, *S*_2_) returns a number between zero and unity that describes the fraction of identical nodes in the two sequences *S*_1_ and *S*_2_. Thus, it has a focus on sequence content and not on sequence position.

Addressing position of nodes in two *s*-core+ sequences *S*_1_ and *S*_2_, we identify nodes (genes) that are central to both sequences by calculating *t*_*i*_(*S*_1_, *S*_2_) for each node *i* (see Eq (2)). Since nodes in the innermost cores will be given the largest values in the core-sequence, *t*_*i*_ will only be near its maximal value for nodes that are in the innermost cores of *both* networks. Similarly, we calculate *u*_*i*_(*S*_1_, *S*_2_) (see Eq (3)) as the difference in node *i*’s sequence position in the two sequences *S*_1_ and *S*_2_. Here, only nodes that are central in one of the two sequences will return *u*_*i*_ near the maximal or minimal value. Thus, by making a density plot of *t*_*i*_ versus *u*_*i*_, we can assess the distribution of genes central to only one or both of the networks.

#### Human all-tissues and brain networks

For the comparison between the *H*_*A*_ and *H*_*B*_ networks, we identify 855 genes common to the two networks, all being TFs since the brain networks only consist of TFs. The 10 genes with the largest *t*_*i*_(*H*_*A*_, *H*_*B*_) values, being most central in both of the networks, are shown in Table 4, along with the corresponding results from the three other network comparisons that will be presented in the subsequent paragraphs. The top-10 genes with the largest *u*_*i*_(*H*_*A*_, *H*_*B*_) and *u*_*i*_(*H*_*B*_, *H*_*A*_) values are shown in Table 5, along with the results from the (*M*_*A*_, *M*_*B*_)-comparison.

**Table 4:** 

*t*(*H*_*A*_, *H*_*B*_)	*t*(*M*_*A*_, *M*_*B*_)	*t*(*H*_*A*_, *M*_*A*_)	*t*(*H*_*B*_, *M*_*B*_)
HOXB8	FOXL1	PCDHB1	SIM2
ESR1	HESX1	PROP1	FEV
DLX4	POU2F3	FPR3	NFATC1
NKX3-1	GCM1	RHO	HOXA11
RUNX2	FOXN1	SLC14A2	NR4A3
TP63	PROX2	BEST2	DLX4
ZNF669	HAND1	PAX4	GATA1
ALX3	FEV	NR4A3	ESR2
NR2E3	NR4A3	OPRM1	NKX3-1
NR1I2	FOXE3	CER1	NR1I2

Genes corresponding to the 10 largest *t*(*S*_1_, *S*_2_)-values (Eq (2)), i.e. the genes that are highly central in both networks, as given by their *s*-core+ decomposition sequences *S*_1_ and *S*_2_. The four columns show (from left to right) the 10 genes that are the most central in both: Human all-tissues and human brain, mouse all-tissues and mouse brain, human all-tissues and mouse all-tissues and human brain and mouse brain. Only TF genes are eligible for these comparisons, except for the third column, *t*(*H*_*A*_, *M*_*A*_), which compares the all-tissues networks.

**Table 5:** 

*u*(*H*_*A*_, *H*_*B*_)	*u*(*H*_*B*_, *H*_*A*_)	*u*(*M*_*A*_, *M*_*B*_)	*u*(*M*_*B*_, *M*_*A*_)
INSM1	RFX7	E130120F12RIK	ZFP768
THRB	ARID3A	NFATC2	MEOX1
SOX10	ARNT	GSX1	RARA
RXRG	ZFP161	ZFP595	ZFP687
YY1	NFATC3	1700012C15RIK	TRP63
PKNOX2	SPI1	DBX1	ESRRA
SOX11	ZSCAN18	ZFP541	BATF3
ZNF236	RUNX3	ZFP352	SOX13
ZFHX4	RFX5	OLIG3	VDR
HOXD1	ZNF134	T	ZFP40

Transcription-factor genes corresponding to the 10 largest, positive *u*_*i*_-values (Eq (3)), i.e. the genes that are most central in the *s*-core+ network decomposition sequence of network *S*_1_ compared to *S*_2_. The four columns show (from left to right) the 10 genes that are the most central in: Human all-tissues compared to human brain, human brain compared to human all-tissues, mouse all-tissues compared to mouse brain and mouse brain compared to mouse all-tissues.

**Fig 2:**
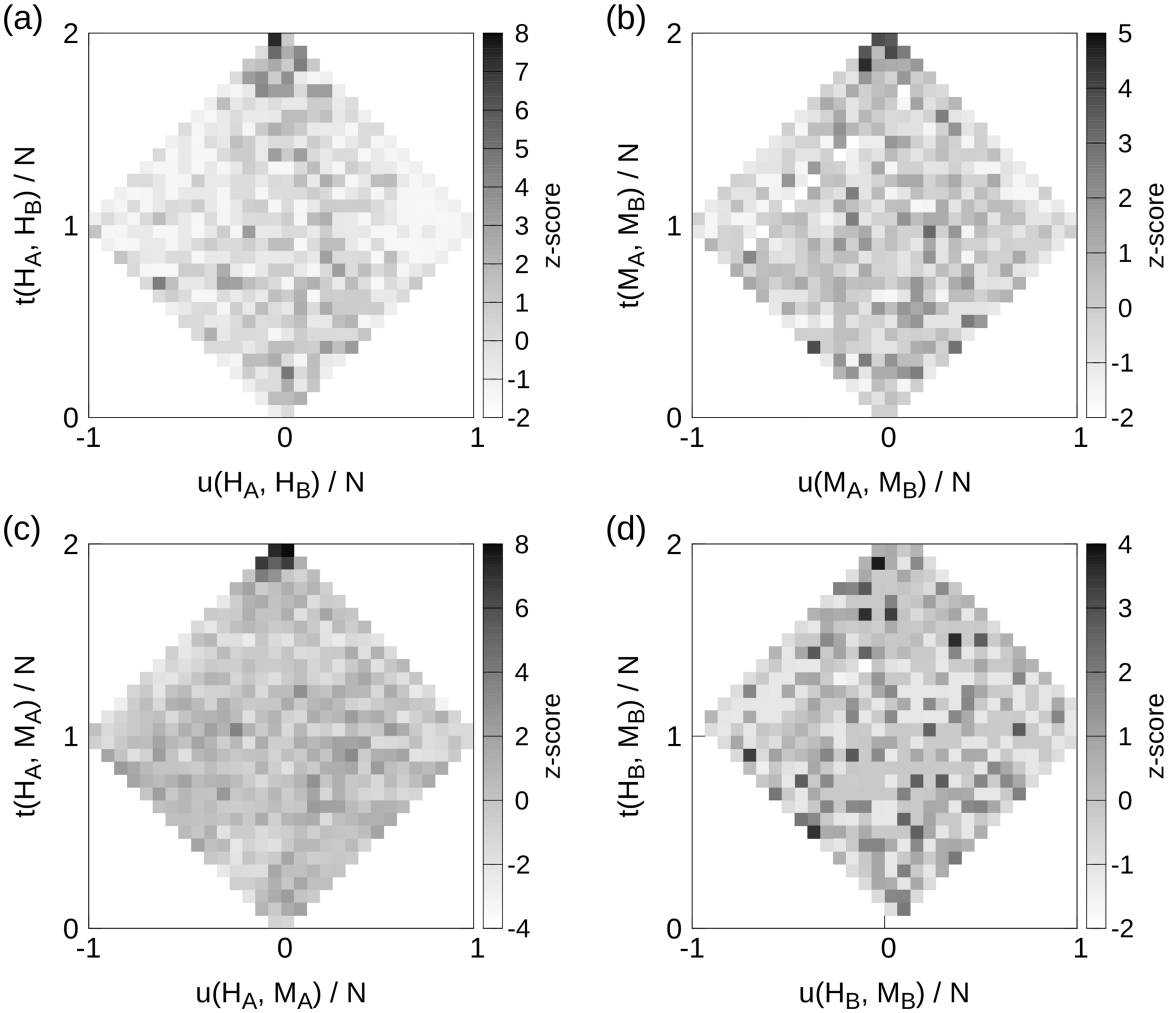
Node density plots of *u*(*S*_1_, *S*_2_) versus *t*(*S*_1_, *S*_2_) for comparisons between the *s*-core+ sequences in: (a) human all-tissues and brain networks, (b) mouse all-tissues and brain networks, (c) human and mouse all-tissues network and (d) human and mouse brain networks. The densities are given by their *z*-score value (see “Methods”). There is a clear statistical over-representation of genes that are central in both *s*-core+ sequence *S*_1_ and *S*_2_ for (a), (b) and (c). The brain-networks comparison in (d) show no clear trend.

**Fig 3:**
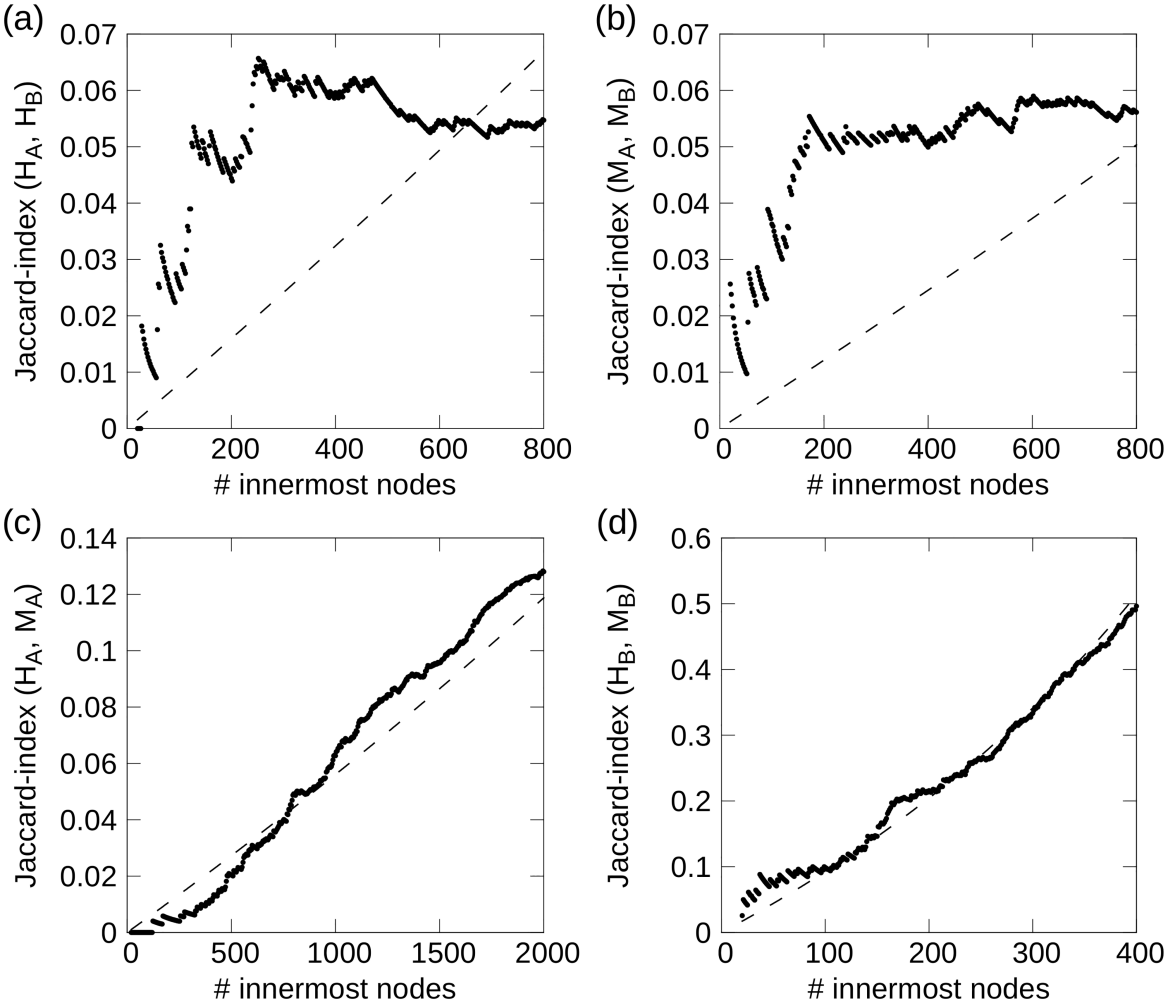
Jaccard index *J*(*S*_1_, *S*_2_) as function of node subsets of *s*-core+ sequence *S*_1_ and *S*_2_ consisting of their *m* respective innermost nodes, for: (a) Human all-tissues and brain, (b) mouse all-tissues and brain, (c) human and mouse all-tissues and (d) human and mouse brain network comparisons. The dashed lines are the expectation value for *J*(*m*; *S*_1_, *S*_2_). Both within-species comparisons show large *J*-values for small *m*, while the within-species comparisons have a lesser extent of innermost core overlap.

The plot of *t*(*H*_*A*_, *H*_*B*_) versus *u*(*H*_*A*_, *H*_*B*_) is shown as a node density plot in Fig 2(a), where the gray-scale show the *z*-score value between the actual density plot and 1000 randomizations (see “Methods”). There is a clear grouping of genes that are central in both the all-tissues and brain networks (large *t*_*i*_-values), aggregated at the top of Fig 2(a). A maximum *z*-score ∼ 8, with a neighborhood of *z* ≥ 4, for large *t*_*i*_-values, demonstrate that many TFs are strongly co-expressed and central in both the human brain and all-tissues network. Another demonstration of this is shown in Fig 3(a), where the Jaccard index *J*(*H*_*A*_(*m*), *H*_*B*_(*m*)) between equally sized subsets of *H*_*A*_ and *H*_*B*_, is plotted as function of the node subset containing the *m* innermost nodes in the respective sequence.

#### Mouse all-tissues and brain networks

Here, there are 1,024 overlapping TF genes, which, like the (*H*_*A*_, *H*_*B*_)-comparison, show larger node densities in the top of Fig 2(b), though with a smaller maximum *z*-score value ∼ 5. From Fig 3(b), we see that there is a relatively high degree of overlap between the innermost *s*-cores in mouse all-tissues and brain networks. This is not that surprising, given the results depicted in Fig 1, where TFs are clearly enriched for the all-tissues networks. The reason for the relatively small *J*-values in Fig 3(a) and 3(b) is that the size of the compared networks are very different, resulting (most likely) in large unions (*M*_*A*_ ∪ *M*_*B*_) compared to intersections (*M*_*A*_ ∩ *M*_*B*_). Since the network cores are very dense, and the overlap is relatively small, intersection network approaches are not suitable for our networks.

#### Human and mouse all-tissues networks

In this network comparison, we are not limited to TFs only, as with the other comparisons, since both the human and mouse all-tissues networks contain all the genes provided by the data sets. Using orthologs as mapping between the two *s*-core+ network decomposition sequences, we find 9,420 overlapping genes. The top-10 genes with the largest *u*_*i*_(*H*_*A*_, *M*_*A*_) and *u*_*i*_(*M*_*A*_, *H*_*A*_) values are shown in Table 5, along with the results from the (*H*_*B*_, *M*_*B*_)-comparison.

The node density plot of the within-species all-tissues network comparison is shown in Fig 2(c). With maximal *z*-scores *z* ∼ 8 in the large-*t* region, this comparison also demonstrate the same behavior as the human and mouse all-tissues and brain comparisons, namely that there is an over-representation of orthologs that are central in both the *s*-core+ decomposition sequence *H*_*A*_ and *M*_*A*_. This result is not apparent given Fig 3(c), but there is a clear segment where the actual overlap is larger than the expected, leading to the increased density of conserved orthologs with large *s*-core index values in both species.

#### Human and mouse brain networks

These two networks consist of TFs only, and as this is an within-species comparison, we are also restricted to ortholog data, resulting in 543 overlapping genes. Fig 2(d) is the single figure in the figure panel not showing an indication towards conservation of innermost orthologs. This is probably caused by this network comparison being the only comparison between two TF-only networks. We also see that the *J*(*H*_*B*_, *M*_*B*_)-index shown in Fig 3(d), follow the random expectations quite closely. It should be noted, however, that this similarity comes largely from the visualization of the *J*-index plot. The other plots are based on the overlap of many thousands of genes, while there are ∼ 700 orthologs in max(|*H*_*B*_|, |*M*_*B*_|). This naturally result in larger *J*-values compared to the other figures.

### TF and gene ontology term enrichment in network comparisons

#### Human all-tissues and brain networks

**Fig 4:**
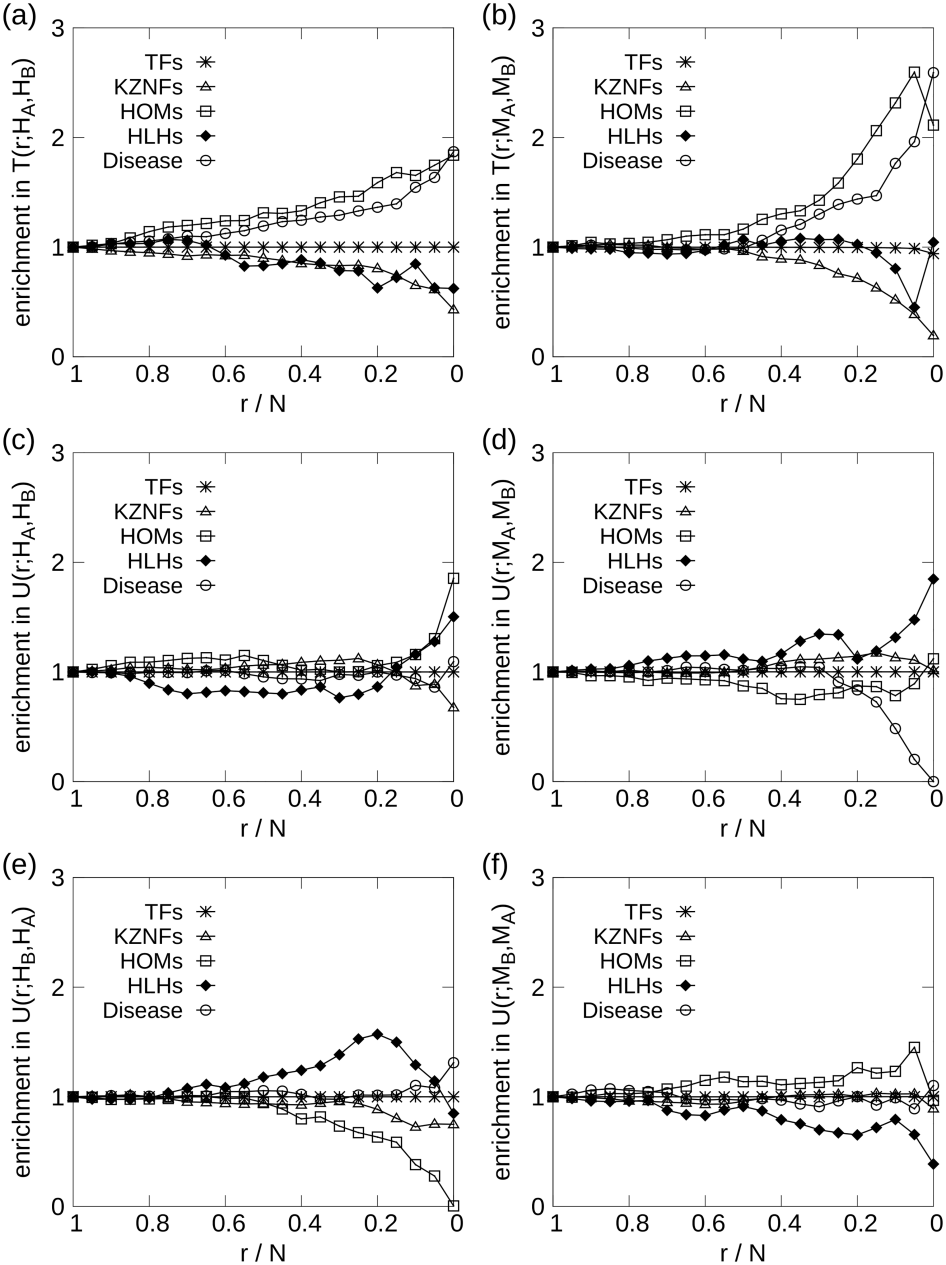
Enrichment of TFs for the human and mouse all-tissues and brain network comparisons. The left column show (*H*_*A*_, *H*_*B*_) comparison, while the right compare (*M*_*A*_, *M*_*B*_). HOMs dominate the *t*_*i*_ enrichment, while being less prominent in the *u*_*i*_ comparisons. HLHs are enriched for (d) and (e). The x-axis is displayed in descending order, since *r* is the number of innermost genes, meaning that *r*/*N* = 1 denote the entire gene set, while smaller *r*/*N* denote gene sets of increasing centrality.

The enrichment of TF families within the node set containing the *r* centralmost genes according to the *s*-core+ sequence comparison measures *t*(*H*_*A*_, *H*_*B*_), *u*(*H*_*A*_, *H*_*B*_) and *u*(*H*_*B*_, *H*_*A*_) are shown in Fig 4(a), 4(c) and 4(e) respectively. In Fig 4(a), we see significant enrichment of HOMs, even for large *r*-values, indicating that certain HOMs have a similar regulatory role in both networks. The 10 genes with the largest *t*_*i*_(*H*_*A*_, *H*_*B*_) values are shown in Table 4, and among them the HOM genes: HOXB8, DLX4, and ALX3.

Fig 4(c) depicts the enrichment of genes that are central in the all-tissues network, but not in the brain network. The comparison of the reversed sequence difference, *u*(*H*_*B*_, *H*_*A*_), is displayed in Fig 4(e), and we observe significant under-representation of HOMs among the ≲30% of the innermost genes. HLHs are enriched among the 30% to 20% nodes that are most central in brain compared to the all-tissues network.

The top-100 nodes that are central in both networks, i.e. *T*(100; *H*_*A*_, *H*_*B*_), are enriched for GO function terms including receptor and transcription factor activity (see S5 Table), the latter of which is an obvious consequence of the TF enrichment among brain nodes. Among the all-tissues centric difference nodes, *U*(100; *H*_*A*_, *H*_*B*_), the GO terms related to vesicles and extracellular exosome were more than 4-fold enriched. For the brain-centric case, *U*(100; *H*_*B*_, *H*_*A*_), GOrilla reported significant enrichment of processes related to cell adhesion.

#### Mouse all-tissues and brain networks

The within-species comparison of the mouse networks, provided TF enrichment results shown in Fig 4(b), 4(d) and 4(f) for *T*(*r*; *M*_*A*_, *M*_*B*_), *U*(*r*; *M*_*A*_, *M*_*B*_) and *U*(*r*; *M*_*B*_, *M*_*A*_) respectively. There are many similarities between Fig 4(a) and 4(b) and HOMs are enriched in the mouse case as well. The enrichment results for the *s*-core sequence differences, Fig 4(d) and 4(f), were not significant.

The GO term enrichment analysis did not provide many results for the mouse network comparisons, with two significantly enriched terms: sequence-specific DNA binding, in *T*(100; *M*_*A*_, *M*_*B*_) and multicellular organismal development.

#### Human and mouse all-tissues networks

**Fig 5:**
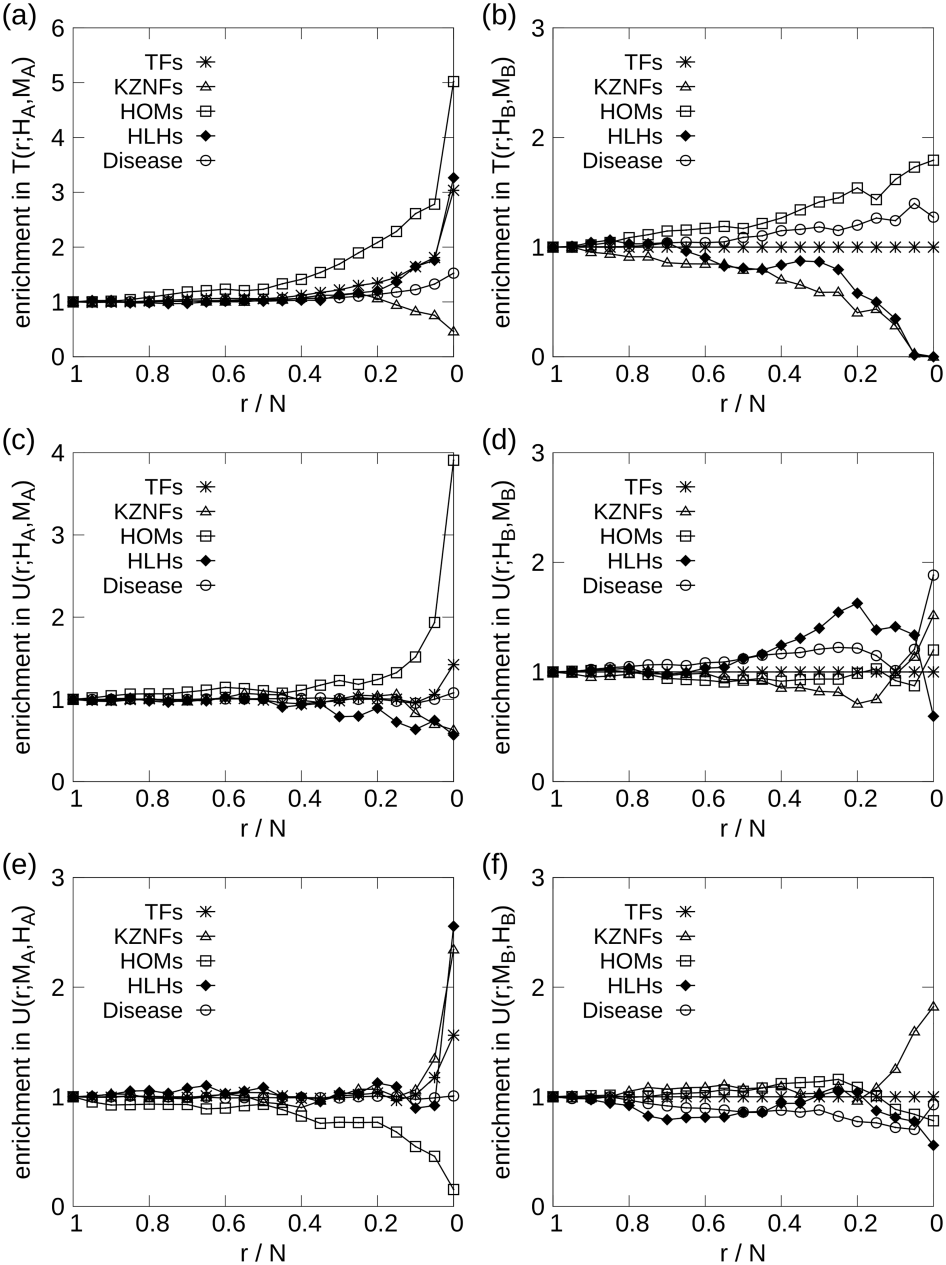
Enrichment of TFs for the all-tissues and brain comparison across species. The left column show (*H*_*A*_, *M*_*A*_) comparison, while the right compare (*H*_*B*_, *M*_*B*_). Note (d), comparison of human and mouse brain network. For the innermost 10% of the genes, KZNFs are enriched in the comparison sequence *u*_*i*_(*H*_*B*_, *M*_*B*_). (e) Enrichment of KZNFs according to *u*_*i*_(*M*_*A*_, *H*_*A*_), which contrasts with (d). The x-axis is displayed in descending order, since *r* is the number of innermost genes, meaning that *r*/*N* = 1 denote the entire gene set, while smaller *r*/*N* denote gene sets of increasing centrality.

For the cross-species comparison between human and mouse all-tissues networks, we are no longer limited to TFs in the enrichment analyses, which can be seen in Fig 5(a), 5(c) and 5(e) depicting the results for *T*(*r*; *H*_*A*_, *M*_*A*_), *U*(*r*; *H*_*A*_, *M*_*A*_) and *U*(*r*; *M*_*A*_, *H*_*A*_) respectively. Fig 5(a) show very high enrichment of TFs, and particularly HOMs, among the genes that are central in both human and mouse all-tissues networks. HLHs are enriched, but the result is barely significant, with *p*-values ∼ 0.01. The enrichment results for the human-centric difference measure, *U*(*r*; *H*_*A*_, *M*_*A*_), shown in Fig 5(c), display highly significant HOM enrichment for *r*/*N* < 0.9, which perhaps is surprising given the individual results for human and mouse displayed in Fig 1(a) and 1(b).

For the mouse-centric comparison, *U*(*r*; *M*_*A*_, *H*_*A*_), Kruppel-type zinc finger TF genes (KZNFs) are significantly enriched for r≲500, while HLHs are enriched for r≲50. HOMs are under-represented, which is not surprising given the human-centric results.

For the GOrilla gene ontology analysis we used the results from the human genes in the within-species analyses, as discussed in this and the following paragraph. There are multiple enriched GO terms for the 1,000 centralmost orthologs, according to the sum measure *T*(1000; *H*_*A*_, *M*_*A*_). The full list of GO function terms are shown in S6 Table, where cytokine activity and receptor activity (with sub-terms) are the most highly enriched terms. Process-wise, GO terms related to sensory perception (*p* = 1.5 ⋅ 10^−12^), G-protein coupled receptor signaling pathway (5.3 ⋅ 10^−9^) and multicellular organismal process (5.3 ⋅ 10^−7^) are the most prominent.

For the human-centric comparison *U*(2000; *H*_*A*_, *M*_*A*_), several enriched sub-terms related to transporter activity were found, as shown in S7 Table. This also led to enrichment of processes related to synaptic signaling (*p* = 7.2 ⋅ 10^−6^). Among the orthologs central in mouse, but not human, *U*(2000; *M*_*A*_, *H*_*A*_), several process terms, including immune system process, cell cycle and DNA metabolic process, were significantly enriched (S8 Table).

#### Human and mouse brain networks

The comparison between human and mouse brain networks comprise the nodes contained in both networks that are both TFs and orthologs. The TF enrichment results, according to the comparison measures *T*(*r*; *H*_*B*_, *M*_*B*_), *U*(*r*; *H*_*B*_, *M*_*B*_) and *U*(*r*; *M*_*B*_, *H*_*B*_), are shown in Fig 5(b), 5(d) and 5(f) respectively. HOMs are enriched, while KZNFs are significantly under-represented for small *r*-values in *T*(*r*; *H*_*B*_, *M*_*B*_). From this, the KZNFs do not show central activity in both networks, but they are central in human brain relative to mouse, as can be seen in Fig 5(b). A significant subset of the KZNF genes, encoding KRAB zinc-finger TFs (KRAB-ZNFs), are known to be rapidly evolving in mammals [32, 33], and their rapid evolution has bee suggested to be related to human brain evolution [8]. The mouse-centric TF enrichment provide no statistically significant results. No highly enriched GO terms were found in the network comparisons.

### Enrichment of disease-associated genes

Similar to the TF and GO term enrichment studies, enrichment experiments were also performed for genes associated with diseases according to OMIM [34] and MGI [35]. The resulting enrichment profiles are shown in Figs 1, 4 and 5. In the entire human all-tissues network, there were identified 2858 disease-associated genes, while 1171 were identified for mouse. In the ortholog comparisons, an orthologous gene is said to be associated with a disease if it is a disease gene in either species; this definition yielded 2543 disease genes in the comparison of the human and mouse all-tissues networks, which consist of 9420 genes in total.

The innermost *s*-cores in the human all-tissue network show significant enrichment for the 1000 innermost genes or so, which can be seen by an upswing on the rightmost side of the curve in Fig 1(a). The same trend appears for the mouse network as well, though not with high statistical significance (*p* ∼ 0.05). For the human brain network, on the other hand, there is highly significant enrichment of disease genes in the innermost cores (*p* ∼ 10^−5^ for the innermost few hundreds of genes), shown in Fig 1(c). A similar trend is seen in the mouse brain network, though, again, with poorer significance.

In Fig 4, the within-species network comparisons are shown, and in both Fig 4(a) and 4(b), for genes central in brain and all-tissues networks, for both human and mouse, we see a clear enrichment of disease-associated genes. The enrichment among the innermost 100 genes in the human within-species comparison, *T*(100; *H*_*A*_, *H*_*B*_), has *p* < 10^−4^, while the corresponding mouse comparison, *T*(100; *M*_*A*_, *M*_*B*_), yields *p* < 10^−3^. The difference comparisons provide no significant results, aside from a clear under-representation of disease genes in the *u*(*M*_*A*_, *M*_*B*_) comparison, with *p* < 0.01, seen in Fig 4(d). This implies that the TFs that are the most central in the mouse all-tissues network relative to the brain network are not associated with disease-related phenotypes in mouse, which can also be seen as a slight, but significant, bulge in Fig 4(e) for the leftmost, peripheral genes.

The across-species comparisons, shown in Fig 5, give some further insight into the role of disease genes in the central parts of the networks. Especially, there is strong enrichment of the orthologous genes that are central in both human and mouse, depicted in Fig 5(a). The curve is deceptively gentle in the rightmost part of the figure, since more than a quarter of all the genes in the comparison are associated with a disease in either mouse or human. For *T*(500; *H*_*A*_, *M*_*A*_), a *p*-value of *p* < 10^−8^ is calculated, and the enrichment stays significant for values larger than 500 as well, even including the innermost 5000 genes. Due to the smaller number of genes in the brain comparisons, Fig 5(b) is more prone to stochasticity, and though the trend is clear, the enrichment only translates to *p*-values of 0.01 < *p* < 0.05 among the centralmost hundred genes, approximately. Fig 5(d) displays significant enrichment for *r*/*N* > 0.2, though no significant enrichment for the innermost 150 genes, even though the curve has a steep ascent as it approaches 0. The same trend is seen for the opposite comparison in Fig 5(e), as an under-representation of disease genes, though these results are statistically dubious with p≳0.95.

## Discussion

In this article, we have applied a novel, data-agnostic network comparison method to gene-expression data from human and mouse. We studied the transcription factor (TF) and gene ontology (GO) term enrichment of single networks and comparisons of networks reconstructed from the data. Networks based on all available tissue types, or from only the brain and central nervous system tissue types, respectively, were used to construct two weighted gene co-expression networks for each species. These four networks were *s*-core+ decomposed into centrality-ranked gene sequences using the *s*-core index as rank parameter, analysed, and subsequently compared within and across species using novel comparison metrics, which are functions of the rank parameters of the compared networks.

The four weighted topological overlap networks, human and mouse, all-tissues and brain and CNS tissues networks, were all very dense and inter-connected, some with average degree of about half of the total number of nodes. After *s*-core+ decomposition, ranked node lists were obtained for the networks. Eqs (2)–(5) were used to compare the centrality of the nodes (genes) in the networks: A direct comparison for the within-species, and through ortholog mapping for the across-species comparison.

TF enrichment analysis of the centralmost genes, and their within- and across-species comparisons, led to the following observations: (1) TFs share co-expression profiles in a highly conserved manner across tissue types for human and mouse, as supported by e.g. Ref. [23]. (2) The homeobox, or HOM, family of TFs are universally co-expressed across tissues and species, which is in concordance with Ref. [25]. (3) KZNF genes are more central in the human brain and CNS network than for the corresponding mouse network. (4) Basic helix-loop-helix TFs are more central in the mouse all-tissues wTO network than in the human counterpart. The GO term enrichment analysis provided several terms that were enriched for both human and mouse all-tissues networks separately, and in particular processes related to regulation and general development: multicellular organismal process, cell-cell signaling and G-protein coupled receptor signaling pathway.

We also note that several of the genes that are among the top-10 centralmost genes in their respective network or network comparison (displayed in Tables 1, 4–6) are ranked top-10 in more than one *s*-core+ sequence or sequence comparison *u* or *t*. For instance, the TF FOXE3 is central in both mouse all-tissues and brain network, and thereby in the comparison *t*(*M*_*A*_, *M*_*B*_) as well. FOXE3 is involved in the regulation of lens epithelial cell growth and is related to the rare eye diseases: anterior segment mesenchymal dysgenesis in human and mouse, and Peters anomaly in mouse (OMIM [34] and MGI [35]). Mutations in FOXO3 also predispose to aortic aneurisms, suggesting a broader role throughout the body [36]. The gene RHO, involved in photoreception, is central in both *H*_*A*_ and *t*(*H*_*A*_, *M*_*A*_), and is also related to eye diseases, including night blindness [34]. RUNX2, ALX3 and RARA are also central in multiple sequences and related to diseases in both human and mouse: cleidocranial dysplasia, frontonasal dysplasia 1 and acute promyelocytic leukemia respectively [34, 35]. Additionally, RARA plays an important role in synaptic transmission and plasticity in the brain [37, 38].

**Table 6:** 

*u*(*H*_*A*_, *M*_*A*_)	*u*(*M*_*A*_, *H*_*A*_)	*u*(*H*_*B*_, *M*_*B*_)	*u*(*M*_*B*_, *H*_*B*_)
ATP6AP2	HSPBAP1	IRF5	CASZ1
STRAP	DSPP	ARNT	GATA4
SYDE1	TNFRSF8	IKZF1	FOXN3
S100G	ID1	ZSCAN12	CUX1
EBF2	MSH5	RUNX2	EGR3
VPS53	RRP7A	RELB	FOXO3
PIGR	PLAG1	HOXB8	BCL6
SOX13	PLCB2	WT1	ZHX3
DLX2	RMI1	HNF1B	ZFP36L2
MMACHC	DCLRE1C	TUB	IRF7

The orthologs corresponding to the 10 largest, positive *u*(*S*_1_, *S*_2_) values (Eq (3)), i.e. the orthologs that are most central in the *s*-core+ network decomposition sequence of network *S*_1_ compared to *S*_2_. The four columns show (from left to right) the 10 orthologs that are the most central in: Human all-tissues compared to mouse all-tissues, mouse all-tissues compared to human all-tissues, human brain compared to mouse brain and mouse brain compared to human brain.

In the mouse brain network, the ALX gene ALX4 was found to be central, which is related to the inherited disorders frontonasal dysplasia 2 and parietal foramina. For the human brain network, TP63, the gene coding for tumor protein p63, is found to be central. It regulates stem cell maintenance, stress response, and tissue repair, and is associated with a number of malformations and syndromes, including cleft lip/palate syndrome, split-hand/foot malformation. TP63 is also associated with a variety of human age-related pathologies [39]. The TRP63 gene, coding for p63 in mouse, is central in the *u*(*M*_*B*_, *M*_*A*_) network comparison, meaning that it is found to be one of the centralmost genes in the mouse brain network, while being one of the most peripheral genes in the mouse all-tissues network. TP63 is also central in the *t*(*H*_*A*_, *H*_*B*_) comparison, i.e. central in both human networks. Genes central in both mouse networks, include FOXN1 and HESX1, related to T-cell immunodeficiency and septo-optic dysplasia respectively. For the human all-tissues network, the genes MC2R and HPSE2 are central and related to diseases: glucocorticoid deficiency and urofacial syndrome respectively. The mouse all-tissues network, on the other hand, provided no diseases related to the top-10 centralmost genes, apart from FOXE3 discussed above.

Among the orthologous genes that are central in the human all-tissues network, but not in the mouse all-tissues network, we found these disease-associated genes: MMACHC, VPS53, DLX2 and ATP6AP2. The latter two of these genes are related to dyslexia and mental retardation and Parkinson’s disease respectively. For the opposite case, *u*(*M*_*A*_, *H*_*A*_), the gene DSPP, related to deafness and tooth abnormalities (dentin dysplasia and dentinogenesis imperfecta) were found alongside PLAG1, DCLRE1C and PLCB2 to be associated with adenomas, severe immunodeficiency and platelet deficiency respectively. For the human-centric brain network comparisons, *u*(*H*_*B*_, *M*_*B*_), the genes IKZF1 and HNF1B, which are related to acute lymphoblastic leukemia and diabetes mellitus type 2 respectively, were found among the centralmost genes. In the brain, IKZF1 is a regulator of pituitary gene expression and lack of this TF leads to widespread dysregulation of glucorticoid signaling, an essential component of whole-organism stress response. For the mouse-centric counterpart, *u*(*M*_*B*_, *H*_*B*_), BCL6 and GATA4 were central genes, associated with B-cell lymphoma and atrioventricular diseases respectively; GATA4 has also recently been linked to stress response and age-related inflammation in multiple tissues including the brain [40].

Another result, which at first appear counter-intuitive, is that the disease genes are enriched in the central cores of many of the networks and network comparisons. Considering the general enrichment of TFs, this might make sense, since it is reasonable that regulatory elements would be associated with a disease, if not directly, then, to some extent, by proxy. Some of the enrichment results can possibly be explained by such mechanisms, e.g. the results shown in Figs 1(a), 1(b) and 5(a). Others may not, such as the results depicted in Fig 1(c), and Fig 4(a) and 4(b) in particular.

Our results suggesting that central, highly connected genes in the network have a strong tendency to be associated with diseases is counter to the more intuitive expectation that disease genes should be localized in the network periphery [41–43]: central genes are highly connected genes, involved in multiple essential functions. However, disease phenotype is not synonymous with null-mutation, and should therefore not be directly correlated to gene essentiality and the known centrality of essential proteins. Rather, our results indicate that highly central genes are more likely to be associated to diseases because they have large influence on the network. Thus, a perturbation of a central gene’s expression profile (ie. not it’s removal) is likely to have non-catastrophic, yet significant and widespread downstream consequences [8]. Our result is in agreement with recent studies suggesting that it is likely for disease genes to occur centrally in the human protein interaction network, because essential disease genes are more highly connected, as well as being more numerous, than the non-essential disease genes [44].

## Conclusion

In this paper, we have developed and applied a novel network comparison approach, applicable to arbitrary network structures and edge-weight distributions, by analyzing gene co-expression networks of human and mouse. The method is based on weighted core-decomposition approaches, and we have demonstrated some of the benefits this methodology has in making comparisons between highly connected complex networks. The methods are widely applicable to other network types, be it from biological or non-biological data, for sparse or dense network, and provides a data-agnostic, parameter-choice minimalistic alternative to established methods. By use of weighted topological overlap and the *s*-core+ core-decomposition method, we found that transcription factors, and homeobox domain transcription factors in particular, display highly similar co-expression profiles in a conserved manner for human and mouse, across tissue types and within and across species. Disease-associated genes were found to be overrepresented among the centralmost genes in the gene co-expression network.

## Methods

We analyzed a published protein-coding transcriptome data set consisting of Affymetrix microarray data from 73 human and 72 mouse tissue types [26]. The human data sets contains expression data from 11896 genes, of which 931 are TFs, whereas the mouse data contain 15720 genes, 1147 of which are TFs. There are 21 different tissue types from brain and central nervous system (CNS) in the human data set and 10 in mouse. There are 9420 confirmed orthologs between human and mouse contained in both data sets [45, 46].

### Gene-correlation analysis

To construct networks from the gene expression data sets, we generated gene-correlation matrices, in which genes sharing similar patterns of expression over the samples, receive large pair-wise correlation values. There exist many mathematical measures that can be used to quantify the co-expression similarity, and we chose to use the Spearman rank correlation coefficient, as it is powerful in discovering trends rather than focusing on numerically similar number sequences.

We created two gene-correlation matrices for each species: a matrix based on the expression data from all tissue types, and a matrix constructed solely from the expression data from the tissue types from brain and central nervous system (21 for human and 10 for mouse). The raw gene-expression data sets are matrices of *N* mRNA probes, a term we will use interchangeably by genes in the following, measuring the gene expression in samples from *T* tissue types. The Spearman correlation values *a*_*ij*_ between node *i* and *j*, which compare the ranked expression similarity of node *i* and *j* over the *T* tissues, are calculated for all node pairs, yielding symmetrical *N* × *N* gene-correlation matrices *A* = [*a*_*ij*_], with *a*_*ij*_ ∈ [−1, 1].

### Weighted gene co-expression network

The resulting networks are large, fully connected (all-to-all) correlation networks, and a means for separating central from un-central nodes is needed. For this, we use the weighted topological overlap (wTO) link-weight measure [8, 12, 13, 47], which robustly integrates the neighborhood-similarity of pairs of connected nodes. Contrary to common practice in weighted gene co-expression network analysis, we proceeded with the wTO analysis without using a cutoff of any kind. We want to use a minimum of assumptions and mathematical manipulations, rather including the weak and medium correlation values in our analysis as they indeed constitute the bulk of the links. Also, hard cutoffs lower the statistical significance of the wTO matrix elements if the correlation matrix is constructed from few samples [12, 48] (as for the mouse brain network based on 10 tissue types). This choice implies the usage of the non-negative version of the wTO metric, since the signed version [8] would break the transitivity between triplets of small correlation values. Then [12, 13],

$$\begin{array}{c} w_{ij}=\frac{\sum_{k}|a_{ik}a_{kj}\left|+\right|a_{ij}|}{\text{min}\left(s_{i},s_{j}\right)+1-\left|a_{ij}\right|}, \end{array}$$ (1)

where *s*_*i*_ = ∑ _*i*_|*a*_*ij*_| is the node strength of node *i*, *s*_*j*_ = ∑ _*j*_|*a*_*ij*_| is the node strength of node *j* and the sum over *k* denotes the shared neighbors of node *i* and *j*, which are all the other nodes in our case. min(*s*_*i*_, *s*_*j*_) is the smallest of the two node-strength values, and *w*_*ij*_ therefore range from 0 to 1.

### Network randomization provide statistically significant cutoff values

Randomization tests of the correlation matrices, with subsequent wTO calculations, were performed in order to find suitable cutoff values for the link-weights in the wTO networks. With *T* = 73 and *T* = 72 tissues for human and mouse respectively in the all-tissues networks, the maximal randomized wTO-values are small (maximal values of *w*_*ij*_ ∼ 0.15) compared to the majority of the link-weights. A conservative cutoff of *w*_cut_ = 0.30, was chosen for these two networks.

For the networks based on brain and CNS tissues, however, there is greater uncertainty in the correlation values due to the smaller number of tissues, increasing the false positive rate. This is particularly problematic for the mouse brain correlation matrix as it is constructed from only 10 tissue types. This problem can partly be circumvented by the use of a smaller set of genes *i* and *j* in Eq (1). We chose *i* and *j* to include all the TFs for the two brain networks, while *k* still sums over all the genes, thus including the information of the neighboring non-TF genes in the TF-to-TF wTO network. For the human brain network, the maximal wTO-values after randomization studies was *w*_*ij*_ ∼ 0.25. For the mouse brain network, however, the maximal generated wTO-value from randomization was *w*_*ij*_ = 0.41. For tens of millions of generated values, we found *p* = 1.5 ⋅ 10^−5^ for *w*_*ij*_ ≥ 0.40. For the brain networks, we therefore chose *w*_cut_ = 0.40, which is conservative for the human case, and reasonable for the mouse brain network.

After applying the cutoffs to the wTO networks, we are left with four robust networks with high statistical power, where similarly co-expressed genes with strong neighborhoods are central in the network. These are the four networks we will analyze and compare, i.e. the human and mouse networks from all tissues or brain and CNS tissues, which will be denoted by the species and “all-tissues” or “brain” for simplicity.

### *s*-core+ network decomposition method

The main analysis tool in the network analyses, was *s*-core+ network decomposition, a weighted core decomposition method [20]. *s*-core [19] is the *k*-core equivalent network decomposition method for weighted networks, whereas *s*-core+ is a further modification of the *s*-core method.

The *s*-core algorithm removes the node with the smallest node strength value from the network, using that value as a threshold for which neighboring nodes are gauged against. If the node strength of any neighboring nodes falls below the gauging threshold, that node, with all corresponding links, is also removed. This continues until no remaining nodes have node strength values below the threshold. The procedure repeats until an innermost *s*-core is reached, meaning that removal of the single weakest node inevitably causes all remaining nodes to fall beneath the threshold, thus collapsing the network.

When an innermost *s*-core is reached, *s*-core+ commences, removing the link with the lowest absolute weight value from the core recursively, until a node strength falls beneath the threshold. This node strength becomes the new threshold value for the *s*-core algorithm, which will run until a new innermost *s*-core is reached (it could be the same set of nodes as the previous innermost core). This alternation between node and link removal continues until every node is removed. The nodes are labeled by an *s*-core index *n*, indicating the last distinct *s*-core they were a part of (analogous to *k* in *k*-core analysis). The *s*-core index *n* serves as a centrality measure, where a small *n*-value relates to low centrality, and thereby low inferred importance in the network, while large *n*-values appear for nodes that are strongly connected to central clusters, and thereby deemed to have high importance in the network. By use of this method, the network is decomposed into indexed cores, providing a list of centrality-ranked nodes. Other methods can be used to provide centrality ranking of nodes in complex weighted networks. Among these, Eigenvector centrality (EVC) [27, 28] is perhaps the most sensible to use for comparison with *s*-core+, as it also favors high-strength nodes that are situated near the topological center(s) of the network. In the Results section, we will briefly compare the two approaches, by also calculating the EVC rank for the four networks. In short, we find an overall high concurrence between the methods. However, the EVC and *s*-core+ methods rank the innermost/centralmost nodes in the network somewhat differently; a trend which is more prominent for the mouse networks than the human counterparts.

In the case of dense and highly interconnected weighted networks, where the degree distribution is dominated by large degrees relative to *N*, the *s*-core network decomposition method is more suitable than the *k*-core decomposition method. Also, if the innermost *s*-cores are large, the link-pruning *s*-core+ method is applied in order to distinguish between the nodes in the innermost *s*-core. We will denote the *s*-core+ sequences for the human all-tissues, human brain, mouse all-tissues and mouse brain network by *H*_*A*_, *H*_*B*_, *M*_*A*_ and *M*_*B*_ respectively.

### Comparative network analysis using *s*-core+ node sequences

We use two different measures when comparing the position of node *i* in two *s*-core+ network decomposition sequences *S*_1_ and *S*_2_ (e.g. *H*_*A*_ and *H*_*B*_): The difference *u*_*i*_ and the sum *t*_*i*_ in core-sequence position of node *i*. Since the sequences *S*_1_ and *S*_2_ do not contain the same number of nodes, in general, we use a normalized version of the *s*-core index *n*, denoted by *n*′, which is the reordered mapping of *n* after only the overlapping nodes between sequence *S*_1_ and *S*_2_ (*S*_1_ ∩ *S*_2_) have been kept. The number of nodes in the intersection of the *s*-core+ sequences *S*_1_ and *S*_2_ is *N*_*S*_1_∩*S*_2__, and *n*′ is normalized so that *n*′ ∈ [1, *N*_*S*_1_∩*S*_2__]. The expression for the sequence sum *t*_*i*_ and the difference *u*_*i*_ for the nodes *i* contained in both sequence *S*_1_ and *S*_2_ is then:

$$\begin{array}{ccc} t_{i}\left(S_{1},S_{2}\right)=t_{i}\left(S_{2},S_{1}\right)=n_{i,S_{1}}^{\prime }+n_{i,S_{2}}^{\prime }, & & t_{i}\in \left[0,2N_{S_{1}\cap S_{2}}\right], \end{array}$$ (2)

$$\begin{array}{ccc} u_{i}\left(S_{1},S_{2}\right)=-u_{i}\left(S_{2},S_{1}\right)=n_{i,S_{1}}^{\prime }-n_{i,S_{2}}^{\prime }, & & u_{i}\in \left[-N_{S_{1}\cap S_{2}},N_{S_{1}\cap S_{2}}\right]. \end{array}$$ (3)

A large, positive *u*_*i*_(*S*_1_, *S*_2_) value, corresponds to a node *i* that is among the innermost *s*-cores in sequence *S*_1_, but among the outermost in sequence *S*_2_. Nodes with large, negative *u*_*i*_(*S*_1_, *S*_2_) values have large *n*-values in the *s*-core+ network decomposition sequence *S*_2_, while small *n*-values in *S*_1_. A large *t*_*i*_-value imply that node *i* is a member of the innermost *s*-cores in both *S*_1_ and *S*_2_, while small *t*_*i*_-values correspond to the opposite.

**Fig 6:**
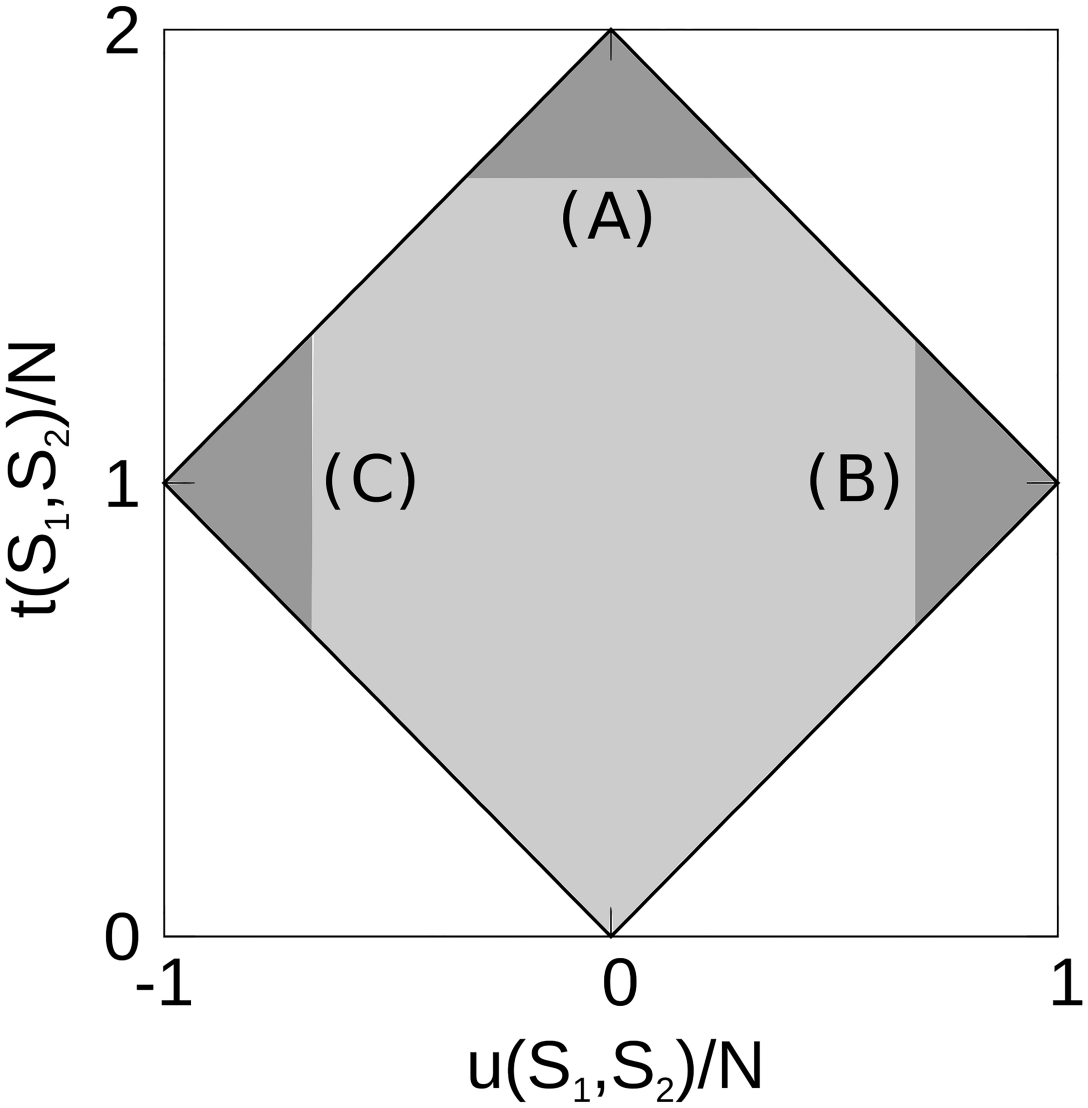
Relationship between *t*(*S*_1_, *S*_2_) and *u*(*S*_1_, *S*_2_) normalized according to the number of nodes *N* = *N*_*S*_1_∩*S*_2__. A node *i* must be within the gray, diamond-shaped area because of the limitations from Eqs (2) and (3). The dark gray areas contain nodes where: (A) $n_{i,S_{1}}^{\prime }$ and $n_{i,S_{2}}^{\prime }$ are large, i.e. the node is central in both *s*-core+ sequences 1 and 2. (B) $n_{i,S_{1}}^{\prime }$ is large, while $n_{i,S_{2}}^{\prime }$ is small, i.e. the node is central in *s*-core+ sequence 1 and peripheral in sequence 2. (C) $n_{i,S_{2}}^{\prime }$ is large, while $n_{i,S_{1}}^{\prime }$ is small, which is the opposite of the (B) criteria, i.e. the node is central in *s*-core+ sequence *S*_2_, but peripheral in sequence *S*_1_.

Fig 6 shows the possible *u*_*i*_(*S*_1_, *S*_2_)/*N*_*S*_1_∩*S*_2__ and *t*_*i*_(*S*_1_, *S*_2_)/*N*_*S*_1_∩*S*_2__ coordinates for any node *i*. For small *t*_*i*_(*S*_1_, *S*_2_)-values, neither $n_{i,S_{1}}^{\prime }$ nor $n_{i,S_{2}}^{\prime }$ can be large, so |*u*_*i*_(*S*_1_, *S*_2_)| is limited to the maximal value of $\text{max}(n_{i,S_{1}}^{\prime },n_{i,S_{2}}^{\prime })$. This is the single limitation of *t*_*i*_ and *u*_*i*_ until *t*_*i*_/*N*_*S*_1_∩*S*_2__ > 1. Then, |*u*_*i*_(*S*_1_, *S*_2_)| is limited by the sum of $n_{i,S_{1}}^{\prime }$ and $n_{i,S_{2}}^{\prime }$. Therefore, we are left with the diamond-shaped box as shown in Fig 6.

To ascertain statistical significance in the node density scatterplots between *t*_*i*_(*S*_1_, *S*_2_) and *u*_*i*_(*S*_1_, *S*_2_), we calculated the *z*-scores (standard scores, *z* = (*x* − *μ*)/*σ*) for the (*t*(*S*_1_, *S*_2_), *u*(*S*_1_, *S*_2_)) node density histograms. The scores were based on 1000 node randomizations on the inherent *s*-core+ sequence structure.

We also apply the Jaccard index as node overlap measure. The Jaccard index is defined as *J*(*S*_1_, *S*_2_) = |*S*_1_ ∩ *S*_2_|/|*S*_1_ ∪ *S*_2_|, where the |…| denote the set cardinality, i.e. number of nodes in the set within the operator.

### Comparative enrichment and gene-ontology analysis

For the human and mouse all-tissues and brain networks, we use the centrality-ranked *s*-core+ sequence to pick subsets of innermost (largest *n*-values) and outermost (smallest *n*-values) nodes to compare. For the comparative networks, we use Eqs (2) and (3) to define node importance in the network comparisons. In Fig 6, this relates to an arrow of increasing node-importance: going from 0 to A along increasing *t*(*S*_1_, *S*_2_)-values, going from C to B along increasing *u*(*S*_1_, *S*_2_)-values, and finally going from B to C, along increasing *u*(*S*_2_, *S*_1_)-values (i.e. decreasing *u*(*S*_1_, *S*_2_)-values).

We are also interested in the number of nodes *r* with the largest *t*_*i*_ or *u*_*i*_ values. E.g. the top 100 nodes, or genes, that are central in human brain, but peripheral in mouse brain. Consequently, we define the node sets:

$$\begin{array}{c} T\left(r;S_{1},S_{2}\right):\text{the}\;r\;\text{nodes}\;\text{with}\;\text{largest}\;t_{i}\left(S_{1},S_{2}\right)\text{-values}\;\text{for}\;\text{all}\;\text{nodes}\;i\in S_{1}\cap S_{2}, \end{array}$$ (4)

$$\begin{array}{c} U\left(r;S_{1},S_{2}\right):\text{the}\;r\;\text{nodes}\;\text{with}\;\text{largest}\;u_{i}\left(S_{1},S_{2}\right)\text{-values}\;\text{for}\;\text{all}\;\text{nodes}\;i\in S_{1}\cap S_{2}. \end{array}$$ (5)

Here, *r* is as a cutoff defining the size of the node subsets *T* and *U*, where *r* ∈ [1, *N*_*S*_1_∩*S*_2__]. We can visualize Eqs (4) and (5) by use of the dark gray triangles A, B and C shown in Fig 6. Assuming that *a* of the nodes are contained in triangle A, *b* in B and *c* in C, then, triangle A envelops the node subset *T*(*a*; *S*_1_, *S*_2_), B envelops *U*(*b*; *S*_1_, *S*_2_) and C envelops *U*(*c*; *S*_2_, *S*_1_). Also note that *U*(*r*; *S*_1_, *S*_2_) = *U*(*N*_*S*_1_∩*S*_2__ − *r*; *S*_2_, *S*_1_).

The enrichment analysis is done using the TF under-groups [49, 50]: KRAB zinc-finger genes (KZNFs), homeobox genes (HOMs) and basic helix-loop-helix genes (HLHs), where enrichment is calculated as: Fraction of under-group in subset divided by fraction of under-group in full set.

For discovering significantly enriched gene ontology (GO) terms in the central gene subsets, we use the web-based gene ontology analysis tool GOrilla [29–31], which is a tool for identifying enriched GO terms related to process, function and component, organizing the results in hierarchies. As we are interested in the big picture, and not only the few innermost nodes, we use gene-subset target sizes of 10% to 20% in general, with default *r*-values of *r* = 100 for the TF-only comparisons, and *r* = 1000 for the all-genes comparisons (*r* from Eqs (2) and (3)). For a term to be considered significant, the Benjamini-Hochberg [51] multiple testing false discovery rate (FDR) corrected *p*-values must provide *p* < 0.05. Unless specified, all *p*-values are Benjamini-Hochberg corrected.

## Supporting information

S1 Fig Scatter plot between *s*-core+ rank and eigenvector centrality rank for human all-tissues network. The rank is sorted so that a rank of 1 is the innermost/centralmost gene.
(PDF)

S2 Fig Scatter plot between *s*-core+ rank and eigenvector centrality rank for mouse all-tissues network. The rank is sorted so that a rank of 1 is the innermost/centralmost gene.
(PDF)

S3 Fig Scatter plot between *s*-core+ rank and eigenvector centrality rank for human brain network. The rank is sorted so that a rank of 1 is the innermost/centralmost gene.
(PDF)

S4 Fig Scatter plot between *s*-core+ rank and eigenvector centrality rank for mouse brain network. The rank is sorted so that a rank of 1 is the innermost/centralmost gene.
(PDF)

S1 Table Top 200 innermost genes in (from left to right) human all-tissues, mouse all-tissues, human brain and mouse brain network. The list is ranked so that rank 1 denote the two innermost genes according to the *s*-core+ sequences. For some of the sequences the gene with rank *i* is contained in the same *s*-core as the gene with rank *i* + 1, so that they actually share rank (2*i* + 1)/2. This degenerative effect is small since most innermost *s*-shells consist of single genes. One exception is the mouse brain network, where there is a cascade between the *s*-core containing 317 genes and the next, containing 171 genes.
(PDF)

S2 Table GO function term enrichment among the 1000 centralmost genes in the human all-tissues network. (PDF)

S3 Table GO function term enrichment among the 1000 central-most genes in the mouse all-tissues network. (PDF)

S4 Table GO function term enrichment among the 200 central-most genes in the human brain network. (PDF)

S5 Table GO function term enrichment according to the sum measure *T*(100; *H*_*A*_, *H*_*B*_) in the human all-tissues and brain network comparison. (PDF)

S6 Table GO function term enrichment according to the sum measure *T*(1000; *H*_*A*_, *M*_*A*_) in the human and mouse all-tissues network comparison. (PDF)

S7 Table GO function term enrichment according to the human-centric difference measure *U*(2000; *H*_*A*_, *M*_*A*_) in the human and mouse all-tissues network comparison. (PDF)

S8 Table GO process term enrichment according to the mouse-centric difference measure *U*(2000; *M*_*A*_, *H*_*A*_) in the human and mouse all-tissues network comparison. (PDF)
