# Supplementary material for: Comparative analysis of weighted gene co-expression networks in human and mouse
Source: PLoS One. 2017 Nov 21;12(11):e0187611. doi: 10.1371/journal.pone.0187611 (PMC5697817; doi:10.1371/journal.pone.0187611)
Supplement: S1 Fig — The rank is sorted so that a rank of 1 is the innermost/centralmost gene. (PDF) [file pone.0187611.s001.pdf]

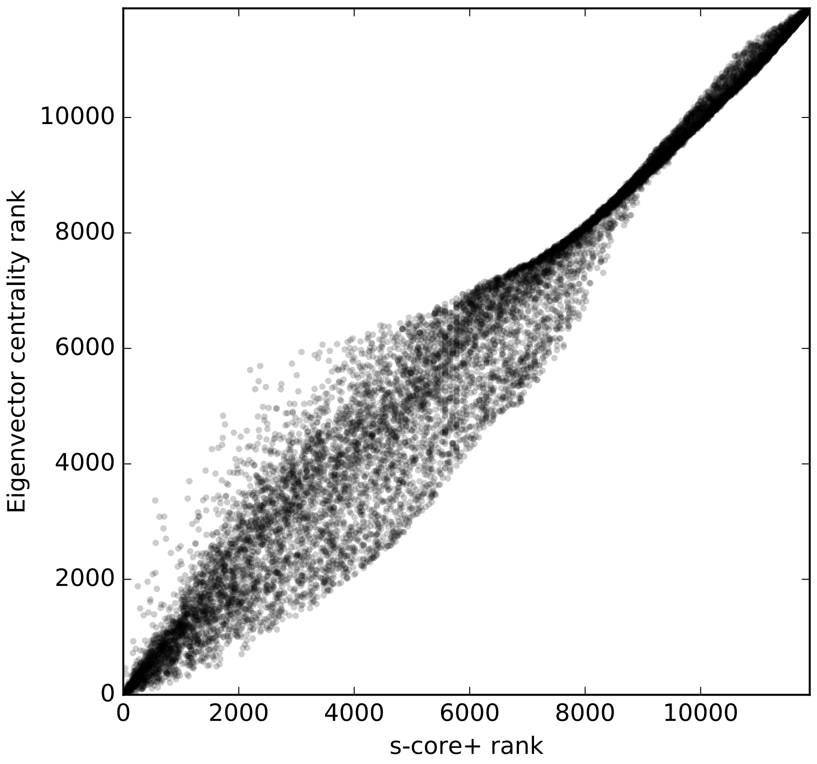

Figure S1. Scatter plot between *s*-core+ rank and Eigenvector centrality rank for human all-tissues network. The rank is sorted so that a rank of 1 is the innermost/centralmost gene.
