# Supplementary material for: Comparative analysis of weighted gene co-expression networks in human and mouse
Source: PLoS One. 2017 Nov 21;12(11):e0187611. doi: 10.1371/journal.pone.0187611 (PMC5697817; doi:10.1371/journal.pone.0187611)
Supplement: S4 Fig — The rank is sorted so that a rank of 1 is the innermost/centralmost gene. (PDF) [file pone.0187611.s004.pdf]

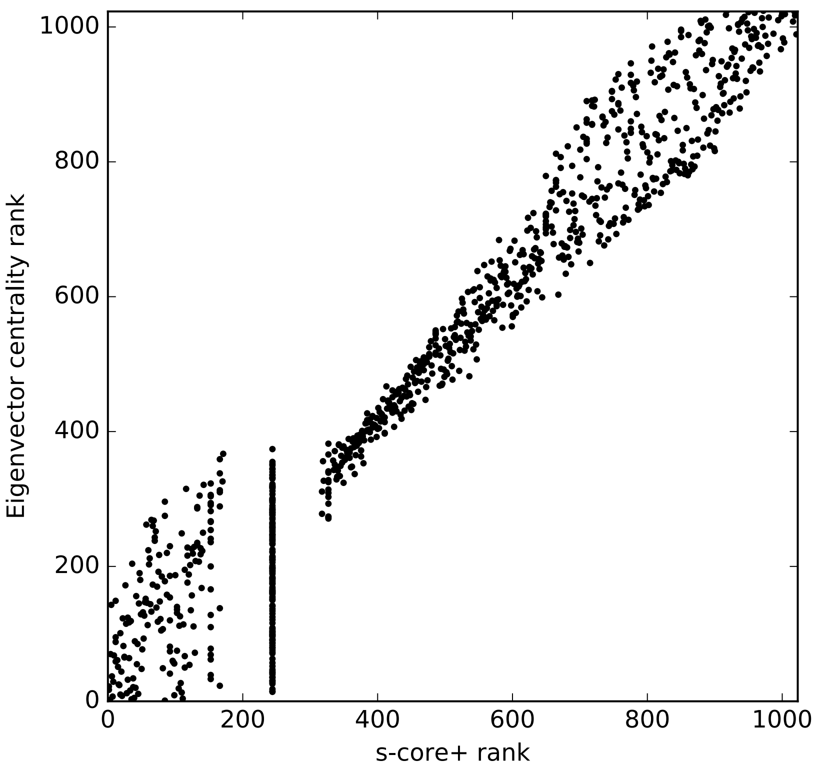

Figure S4. Scatter plot between s-core+ rank and Eigenvector centrality rank for mouse brain network. The rank is sorted so that a rank of 1 is the innermost/centralmost gene.
