# Supplementary material for: Comparative analysis of weighted gene co-expression networks in human and mouse
Source: PLoS One. 2017 Nov 21;12(11):e0187611. doi: 10.1371/journal.pone.0187611 (PMC5697817; doi:10.1371/journal.pone.0187611)
Supplement: S1 Table — The list is ranked so that rank 1 denote the two innermost genes according to the s-core+ sequences. For some of the sequences the gene with rank i is contained in the same s-core as the gene with rank i + 1, so that they actually share rank (2i + 1)/2. This degenerative effect is small since most innermost s-shells consist of single genes. One exception is the mouse brain network, where there is a cascade between the s-core containing 317 genes and the next, containing 171 genes. (PDF) [file pone.0187611.s005.pdf]

Table S1

Rank	H_A	M_A	H_B	M_B
1	MTMR8	IL2	SPIB	HAND1
1	OPRM1	FOXE3	TFAP2A	ZFP36L2
3	RUNX2	WFIKKN1	IRF4	ZFP40
4	GML	PAG1	ALX3	ZFP41
5	MC2R	ZFP628	TP63	SIM2
6	FAM55D	4931428L18RIK	NKX3-1	FOXE3
7	HTR4	IFNA9	ARNT	ALX4
8	NOX1	UBL4B	NR1I2	POU2F3
9	RHO	ZSCAN10	ZNF669	FEV
10	HPSE2	PAX9	DLX4	RARA
11	BMP15	DMPK	NFATC1	FOXN1
12	GPX5	IL9	NR2E3	NR4A3
13	ADAM22	C79127	PAX9	ZFP474
14	NOS1	PROX2	ESR1	IKZF2
15	CA5B	TMC2	ZNF81	FLI1
16	GFRA4	IFNB1	TBX6	4930432O21RIK
17	HTR1F	ETV2	NRF1	HIF3A
18	PADI3	GJA10	PRDM1	TCFEB
19	EFNA5	ELK4	HOXD13	RFXANK
20	TACR1	CCL1	ZNF257	NKX2-3
21	ADRA1A	IFNA4	FOXA2	HSF2
22	PEX5L	PTCRA	HOXB8	BATF3
23	KCNB2	TAAR1	SCAND2	PAX3
24	CER1	SLC45A2	RUNX3	IRX4
25	GRM8	KIS2	GATA1	GATA4
26	CCRN4L	CABP5	ESR2	HOXC13
27	SLC10A1	KRTAP11-1	PBX2	TRP73
28	EDA	ARD1B	RUNX2	GATA5
29	GRPR	V1RD3	GCM2	DMBX1
30	AFF2	ANKRD36	TBX10	ESR2
31	CHRM5	FOXB2	PPARG	MRPS17
32	ZNF614	N/A	MNX1	HOXB13
33	HTR3B	CYP7A1	VDR	SOX30
34	BMPR1B	TCL1B4	RFX5	GCM1
35	CASP10	SPAG4L	EN2	BARHL2
36	ELK1	MYCS	ELF3	HESX1
37	SLC17A4	KRTAP4-6	NEUROD4	FOXN3
38	MPL	V1RA9	HOXB9	ZBTB5
39	MAGEC3	FOXL1	ZNF407	EVX1
40	TNFSF18	1700027D21RIK	ZSCAN18	ZFP526
41	APOL6	TNFRSF8	RARG	ZBTB7B
42	HCRTR2	AU017455	ZEB2	NR1I2
43	GABRA3	BARHL1	PHOX2A	VAX1
44	DAB1	PXT1	TUB	TEAD1
45	UMOD	INSM2	ASH1L	NFATC4
46	WISP1	KRTAP16-4	ZNF134	NKX2-9
47	DCC	H2-M10.1	IKZF4	MSX1
48	HNRNPK	4930549C01RIK	IRX5	ZFP385A
49	IL21	OC90	ZNF589	NKX3-1
50	PRO1768	PROP1	ETV3	ESRRG

51	KRT12	1700025D03RIK	NHLH1	ZFP646
52	BEST2	GPRC2A-RS5	HOXD3	FOXM1
53	CAPN9	NR4A3	WT1	PROX2
54	GABRB2	LHX4	NFYA	HOXA11
55	PDCD6	OPRM1	HOXA11	TBX20
56	NR2E3	BOLL	FOSL2	LYL1
57	GLP1R	IL1F10	HNF1B	ZFP691
58	DCHS2	RP23-212C14.7	ZNF614	PAX6
59	C11ORF58	PITX3	ZNF835	AIRE
60	FMO6P	NKX2-5	SMAD5	GSC2
61	FPR3	TESP2	PRRX2	TCF15
62	GABRR2	KRTAP5-5	ELK1	LCORL
63	PRKG1	OTTMUSG00000005523	HOXC11	PAX5
64	KCNJ1	CD40LG	RELB	PAX1
65	MBTPS2	PRSS29	SPI1	ESRRB
66	CRNN	VSX1	GFI1	MEF2D
67	FLJ21075	SLC16A8	ZNF324B	ESRRA
68	MDM2	KLRA21	ZSCAN12	BC049807
69	SLC17A1	GSX1	ATF7	CRX
70	GHRHR	MC1R	TFAP2B	ARID3A
71	GABRA6	ZFP69	ZNF747	FOXL1
72	PTP4A2	HES3	ZNF20	GATA1
73	CTNNA3	CER1	HOXD11	MAZ
74	ZNF280A	MRGPRH	ALX1	CUX1
75	DCT	DNAJC5G	ADNP	ZFP316
76	IL1RAPL2	STRC	POU6F2	EGR3
77	ALOXE3	4921511H03RIK	ERG	ZFP398
78	KLK13	4930504O13RIK	ZNF343	6430526N21RIK
79	PCDHB1	NPHS1AS	POU3F1	ZFP579
80	PTGES3	EAR11	PAX4	LHX3
81	KCNJ13	TRIM60	TP73	NKX6-1
82	ZKSCAN3	RPUSD2	HOXA9	CDX2
83	HRG	V1RD9	MYOG	ZSCAN2
84	PDE11A	4933405L10RIK	ZNF550	HOXC9
85	HOXC13	PTGER1	ZNF3	REST
86	GCNT4	1700065D16RIK	PKNOX1	PAX8
87	CHRM2	FKBP6	ZNF574	TBX5
88	NR4A3	NXF2	ZNF480	ZFP282
89	INO80D	KRTAP5-4	TBX5	NKX2-5
90	MMP19	NHLH1	ZNF192	PROP1
91	LUZP2	NHEDC1	TEAD1	DLX4
92	CYP2C9	IL24	ZNF155	PITX1
93	MPP3	CDH26	NR4A3	PITX3
94	CCL16	TBX19	TFAP4	NKX2-6
95	SLC14A2	WNT2B	PDX1	BCL6
96	CLCA1	C86695	PGR	VDR
97	GNAT2	TAS1R1	HESX1	ERG
98	TRPC3	KRTAP26-1	NFATC4	DLX3
99	SERPINB4	AU015228	ZNF816A	BARX1
100	TAS2R4	KRTAP16-10	ZNF549	KLF5
101	PAX4	VAX2OS1	SOX9	ZFP787
102	SLC6A6	TAS1R2	SIX1	GSC

103	CRISP1	BC048562	GCM1	DMRT3
104	EPB41L4A	ASB16	PRDM14	SPDEF
105	CNGB3	GLRA4	HOXD10	NFATC1
106	PDE1C	IRGC1	STAT6	LMX1A
107	UBE2E1	D19ERTD386E	FEV	IRF7
108	CHST5	MASP2	SMAD9	TCFAP2A
109	NR1I2	IMPG1	HEYL	A630033E08RIK
110	ZNF669	MS4A13	ZNF35	RBPJL
111	LECT2	ADORA3	IRX4	HOXB9
112	NAT2	GUCY2E	FOXP3	ZFP768
113	CENPJ	BARX1	NEUROG1	ZFP592
114	EBF2	TRAM2	AIRE	TCF12
115	ANXA13	4931432M23RIK	ELF5	TRP63
116	PRDM14	IL20	E2F4	ETV4
117	PSMD7	4933427D06RIK	SIM2	ZHX3
118	TSHB	EG214321	ATF6B	PAX4
119	ZNF747	1700042G07RIK	HNF4G	LHX5
120	ATXN8OS	IFITM7	ZKSCAN3	TBX1
121	KHDRBS2	CRX	HOXC13	SOX13
122	PDE4C	4933434I20RIK	IKZF1	SOX15
123	ALX3	PRAMEL1	HNF4A	GLIS1
124	HECW1	CDRT4	RUNX1	TCF25
125	AP4E1	FEV	NR1H4	TCFE2A
126	TAS2R1	CIB4	NR5A1	ZIC2
127	TM7SF4	BPIL1	ASCL3	VSX2
128	LOC26102	P2RY4	ELK3	ZSCAN20
129	PROX1	NKX1-2	HOXA1	ZFP277
130	DSCR4	4933421I07RIK	GATA3	ZFP687
131	TLL2	FOXO3	NFATC3	MYF6
132	HNRNPM	MSGN1	ZNF839	ZFP568
133	HOXA2	SP6	RORA	ZFP69
134	IMPG2	TTLL4	HOXB13	CDX1
135	KCNH4	PAX4	NFKB2	ZFP758
136	OTC	ACSBG2	ZNF214	TCFAP4
137	CNGA3	T	ABCA11P	MYBL1
138	DLX2	PDX1	TBX21	ZIC4
139	TBX5	CCDC150	RBPJL	ZFP770
140	TNMD	AYM1	ZNF750	ZSCAN10
141	GJD2	TRPD52L3	ELK4	EOMES
142	GUCA2A	HSFY2	NR5A2	FOXH1
143	RASGRF1	OPN1SW	TP53	KLF1
144	MASP1	GCM1	TLX1	HOXA13
145	S100G	TKTL2	SP140L	SP6
146	IL2RA	RMI1	POU4F1	MEOX1
147	KIAA1772	H2-EA	STAT2	ZFP219
148	GRIK3	FOXK1	SRY	VAX2
149	CNGA1	IFNA5	SP1	TEAD3
150	MAS1	NPFFR2	HOXC8	ELF3
151	HCG4P6	ZFP473	NR6A1	NR5A2
152	SIM2	GLT6D1	TFDP2	IRF3
153	MSR1	HESX1	RARA	PLAGL2
154	TP63	4921504E06RIK	PITX3	ISX

155	THSD4	SEPT1	ZNF37B	YY1
156	NDST4	EVX2	ZFX	ZFP111
157	TBCA	EG624855	ZBTB39	SOX17
158	CYP3A4	TRP53RK	SOX14	ZFP109
159	C10ORF68	LHX3	TWIST1	ATOH7
160	SLC10A2	ACTR5	CDX1	HLX
161	ST7L	FHL3	GATA6	AFF3
162	IL5RA	DENND1B	ZBTB25	LHX1
163	P2RX6	MIP	SOX21	HOMEZ
164	SLC5A7	TRPM1	ZNF639	TCFAP2C
165	NPY6R	TDPOZ1	ZFP161	TCFAP2B
166	RORB	KRT31	SRF	MSX2
167	CHRNA5	CCR8	MYOD1	DMRTC2
168	CEACAM7	DSPP	MNT	E2F4
169	GLRA2	BC018473	IRF5	ZFP369
170	FSTL4	1700011F03RIK	TEAD3	OVOL1
171	ABCB11	SERPINA11	ARID3A	FOXO3
172	RASSF9	KLK1B1	OVOL1	-
173	PRKAB2	OTTMUSG00000015762	GSC2	-
174	ATP5F1	TESP1	TCF12	-
175	AMBN	DUSP21	NEUROG3	-
176	ATP5E	2410004A20RIK	REST	-
177	KRT76	2310079F23RIK	CRX	-
178	BTN2A3	1700058C13RIK	GATA2	-
179	GDF9	TLR9	GLI2	-
180	NEUROD4	PTH2	ZNF154	-
181	NEU3	HYAL5	ZNF141	-
182	KRT75	CLDN16	ZNF132	-
183	SIRT4	SPEM1	ZNF552	-
184	PROP1	CDX4	HOXD9	-
185	KCNMB2	TRIM42	ZNF200	-
186	HYDIN	SLC14A2	RFX7	-
187	NRP2	4932438H23RIK	ZNF385D	-
188	GNRHR	APOBEC4	TBX4	-
189	ODAM	4930511I11RIK	BATF3	-
190	DENR	AKP5	MAFK	-
191	MMP27	GZMN	ZSCAN5A	-
192	CYP4A11	MTL5	MYF5	-
193	UPK1A	FASL	FOXD2	-
194	HNRNPR	1700073E17RIK	HEY1	-
195	PRLR	FRAT1	FLI1	-
196	PGLYRP4	PLA2G2C	ZNF391	-
197	ASCL3	PFN3	SALL1	-
198	KLK10	HAND1	ZNF219	-
199	GRIN2B	RBP3	ZNF787	-
200	CD1B	ZSWIM2	MEF2D	-

Table S1. Top 200 innermost genes in (from left to right) human all-tissues, mouse all-tissues, human brain and mouse brain network. The list is ranked so that rank 1 denote the two innermost genes according to the s-core+ sequences. For some of the sequences the gene with rank i is contained in the same s-core as the gene with rank $i+1$, so that they actually share rank $(2i+1)/2$. This degenerative effect is small since most innermost s-shells consist of single genes. One exception is the mouse brain network, where there is a cascade between the s-core containing 317 genes and the next, containing 171 genes.
