# Supplementary material for: Comparative analysis of weighted gene co-expression networks in human and mouse
Source: PLoS One. 2017 Nov 21;12(11):e0187611. doi: 10.1371/journal.pone.0187611 (PMC5697817; doi:10.1371/journal.pone.0187611)
Supplement: S2 Table — (PDF) [file pone.0187611.s006.pdf]

Table S2

GO Term	Description	FDR p-value	Enrichment
GO:0060089	molecular transducer activity	9.28E-13	1.91
GO:0004872	receptor activity	1.39E-12	1.91
GO:0099600	transmembrane receptor activity	2.05E-12	2.10
GO:0038023	signaling receptor activity	5.51E-11	1.97
GO:0004888	transmembrane signaling receptor activity	2.37E-10	2.01
GO:0005216	ion channel activity	6.70E-10	2.55
GO:0022838	substrate-specific channel activity	8.61E-10	2.51
GO:0015267	channel activity	2.35E-9	2.40
GO:0015276	ligand-gated ion channel activity	2.39E-9	3.58
GO:0022803	passive transmembrane transporter activity	2.58E-9	2.40
GO:0022834	ligand-gated channel activity	2.69E-9	3.58
GO:0004930	G-protein coupled receptor activity	4.98E-9	2.45
GO:0022836	gated channel activity	1.45E-8	2.59
GO:0015075	ion transmembrane transporter activity	2.85E-8	1.92
GO:0008324	cation transmembrane transporter activity	5.38E-8	2.07
GO:0005230	extracellular ligand-gated ion channel activity	1.05E-7	4.40
GO:0022891	substrate-specific transmembrane transporter activity	2.05E-7	1.83
GO:0030594	neurotransmitter receptor activity	2.41E-7	4.07
GO:0046873	metal ion transmembrane transporter activity	3.56E-7	2.26
GO:0022890	inorganic cation transmembrane transporter activity	4.98E-7	2.09
GO:0022857	transmembrane transporter activity	5.95E-7	1.76
GO:0005261	cation channel activity	5.96E-7	2.49
GO:0004871	signal transducer activity	8.51E-7	1.61
GO:0015077	monovalent inorganic cation transmembrane transporter activity	3.42E-6	2.29
GO:0070405	ammonium ion binding	2.32E-5	3.88
GO:0008528	G-protein coupled peptide receptor activity	6.43E-5	3.01
GO:0022892	substrate-specific transporter activity	7.29E-5	1.59
GO:0001653	peptide receptor activity	7.32E-5	2.98
GO:0005125	cytokine activity	1.72E-4	2.44
GO:0005215	transporter activity	1.89E-4	1.53
GO:0005249	voltage-gated potassium channel activity	3.69E-4	3.39
GO:0015081	sodium ion transmembrane transporter activity	4.17E-4	2.71
GO:0030553	cGMP binding	5.31E-4	6.89
GO:0005231	excitatory extracellular ligand-gated ion channel activity	6.57E-4	3.92
GO:0022843	voltage-gated cation channel activity	7.59E-4	2.82
GO:0005244	voltage-gated ion channel activity	9.49E-4	2.43
GO:0022832	voltage-gated channel activity	9.76E-4	2.43

GO:0015103	inorganic anion transmembrane transporter activity	1.17E-3	2.73
GO:0005267	potassium channel activity	1.19E-3	2.81
GO:0046906	tetrapyrrole binding	1.33E-3	2.64
GO:0004222	metalloendopeptidase activity	1.55E-3	2.84
GO:0015079	potassium ion transmembrane transporter activity	1.68E-3	2.54
GO:0005253	anion channel activity	1.81E-3	3.10
GO:0005222	intracellular cAMP activated cation channel activity	1.99E-3	10.04
GO:0004175	endopeptidase activity	2.02E-3	1.86
GO:0015296	anion:cation symporter activity	2.04E-3	3.73
GO:0004890	GABA-A receptor activity	2.41E-3	5.67
GO:0099589	serotonin receptor activity	2.55E-3	6.49
GO:0016712	oxidoreductase activity, acting on paired donors, with incorporation or reduction of molecular oxygen, reduced flavin or flavoprotein as one donor, and incorporation of one atom of oxygen	3.85E-3	5.36
GO:0015294	solute:cation symporter activity	4.44E-3	2.78
GO:0022853	active ion transmembrane transporter activity	4.51E-3	2.48
GO:0019825	oxygen binding	4.71E-3	3.90
GO:0022835	transmitter-gated channel activity	4.87E-3	3.61
GO:0022824	transmitter-gated ion channel activity	4.96E-3	3.61
GO:0030551	cyclic nucleotide binding	5.23E-3	4.15
GO:0016917	GABA receptor activity	5.40E-3	5.07
GO:0008509	anion transmembrane transporter activity	6.46E-3	1.98
GO:0008188	neuropeptide receptor activity	6.67E-3	4.02
GO:0020037	heme binding	6.70E-3	2.51
GO:0008227	G-protein coupled amine receptor activity	6.78E-3	4.02
GO:0070330	aromatase activity	6.82E-3	5.62
GO:0004497	monooxygenase activity	1.02E-2	2.75
GO:0099094	ligand-gated cation channel activity	1.04E-2	2.75
GO:0015464	acetylcholine receptor activity	1.09E-2	4.59
GO:0005221	intracellular cyclic nucleotide activated cation channel activity	1.12E-2	7.53
GO:0043855	cyclic nucleotide-gated ion channel activity	1.14E-2	7.53
GO:0005223	intracellular cGMP activated cation channel activity	1.38E-2	9.64
GO:0036002	pre-mRNA binding	1.50E-2	4.38
GO:0005254	chloride channel activity	1.57E-2	2.86
GO:0005044	scavenger receptor activity	1.75E-2	3.87
GO:0015370	solute:sodium symporter activity	1.76E-2	3.31
GO:0015108	chloride transmembrane transporter activity	2.54E-2	2.62
GO:0008395	steroid hydroxylase activity	2.77E-2	4.02
GO:0015293	symporter activity	2.84E-2	2.29
GO:0004995	tachykinin receptor activity	3.19E-2	12.05

GO:0016709	oxidoreductase activity, acting on paired donors, with incorporation or reduction of molecular oxygen, NAD(P)H as one donor, and incorporation of one atom of oxygen	3.57E-2	3.26
GO:0005217	intracellular ligand-gated ion channel activity	3.58E-2	3.86
GO:0051378	serotonin binding	3.61E-2	6.02
GO:0033038	bitter taste receptor activity	3.66E-2	6.02
GO:0043176	amine binding	3.71E-2	6.02

Table S2. GO function term enrichment among the 1000 centralmost genes in the human all-tissues network.
