# Supplementary material for: Comparative analysis of weighted gene co-expression networks in human and mouse
Source: PLoS One. 2017 Nov 21;12(11):e0187611. doi: 10.1371/journal.pone.0187611 (PMC5697817; doi:10.1371/journal.pone.0187611)
Supplement: S3 Table — (PDF) [file pone.0187611.s007.pdf]

Table S3

GO Term	Description	FDR p-value	Enrichment
GO:0004930	G-protein coupled receptor activity	4.98E-9	2.82
GO:0043565	sequence-specific DNA binding	2.08E-6	1.85
GO:0000981	RNA polymerase II transcription factor activity, sequence-specific DNA binding	1.45E-5	1.96
GO:0004888	transmembrane signaling receptor activity	3.06E-5	1.92
GO:0099600	transmembrane receptor activity	5.98E-5	1.86
GO:0005125	cytokine activity	2.65E-4	2.85
GO:0038023	signaling receptor activity	2.85E-4	1.77
GO:0005132	type I interferon receptor binding	5.09E-4	15.99
GO:0003700	transcription factor activity, sequence-specific DNA binding	6.07E-4	1.64
GO:0001071	nucleic acid binding transcription factor activity	6.74E-4	1.64
GO:0008236	serine-type peptidase activity	6.91E-4	2.67
GO:0004252	serine-type endopeptidase activity	8.53E-4	2.80
GO:0017171	serine hydrolase activity	9.08E-4	2.62
GO:0060089	molecular transducer activity	2.73E-3	1.58
GO:0004872	receptor activity	2.93E-3	1.58
GO:0004871	signal transducer activity	5.66E-3	1.52
GO:0003677	DNA binding	6.05E-3	1.38
GO:0008528	G-protein coupled peptide receptor activity	6.69E-3	2.87
GO:0008527	taste receptor activity	1.02E-2	9.99
GO:0001653	peptide receptor activity	1.03E-2	2.76
GO:0000977	RNA polymerase II regulatory region sequence-specific DNA binding	1.25E-2	1.71
GO:0001012	RNA polymerase II regulatory region DNA binding	1.63E-2	1.69
GO:0009881	photoreceptor activity	1.73E-2	8.88
GO:0044212	transcription regulatory region DNA binding	1.74E-2	1.57
GO:0000975	regulatory region DNA binding	1.74E-2	1.56
GO:0001067	regulatory region nucleic acid binding	1.90E-2	1.56
GO:0000976	transcription regulatory region sequence-specific DNA binding	2.17E-2	1.62
GO:0001228	transcriptional activator activity, RNA polymerase II transcription regulatory region sequence-specific binding	2.61E-2	1.84
GO:0005126	cytokine receptor binding	2.69E-2	2.05
GO:0030021	extracellular matrix structural constituent conferring compression resistance	3.38E-2	15.99
GO:1990837	sequence-specific double-stranded DNA binding	3.48E-2	1.58
GO:0030345	structural constituent of tooth enamel	3.50E-2	15.99
GO:0005212	structural constituent of eye lens	3.54E-2	5.09
GO:0008188	neuropeptide receptor activity	3.62E-2	4.00
GO:0015347	sodium-independent organic anion transmembrane transporter activity	3.91E-2	7.27

Table S3. GO function term enrichment among the 1000 central-most genes in the mouse all-tissues network.
