# Supplementary material for: Comparative analysis of weighted gene co-expression networks in human and mouse
Source: PLoS One. 2017 Nov 21;12(11):e0187611. doi: 10.1371/journal.pone.0187611 (PMC5697817; doi:10.1371/journal.pone.0187611)
Supplement: S4 Table — (PDF) [file pone.0187611.s008.pdf]

Table S4

GO Term	Description	FDR p-value	Enrichment
GO:0043168	anion binding	8.23E-3	2.92
GO:0036094	small molecule binding	1.24E-2	2.77
GO:0001047	core promoter binding	1.36E-2	1.84
GO:0001046	core promoter sequence-specific DNA binding	1.38E-2	1.91
GO:0008289	lipid binding	1.56E-2	2.64
GO:0043565	sequence-specific DNA binding	1.56E-2	1.17
GO:0003682	chromatin binding	1.62E-2	1.56
GO:0044877	macromolecular complex binding	2.28E-2	1.49
GO:0005524	ATP binding	3.17E-2	4.27
GO:0000981	RNA polymerase II transcription factor activity, sequence-specific DNA binding	3.34E-2	1.21
GO:0060089	molecular transducer activity	3.45E-2	1.78
GO:0030554	adenyl nucleotide binding	3.49E-2	4.27
GO:0004871	signal transducer activity	3.73E-2	1.78
GO:0032559	adenyl ribonucleotide binding	3.88E-2	4.27

Table S4. GO function term enrichment among the 200 central-most genes in the human brain network.
