# Supplementary material for: Comparative analysis of weighted gene co-expression networks in human and mouse
Source: PLoS One. 2017 Nov 21;12(11):e0187611. doi: 10.1371/journal.pone.0187611 (PMC5697817; doi:10.1371/journal.pone.0187611)
Supplement: S5 Table — (PDF) [file pone.0187611.s009.pdf]

Table S5

GO Term	Description	FDR p-value	Enrichment
GO:0004879	RNA polymerase II transcription factor activity, ligand-activated sequence-specific DNA binding	3.58E-2	2.97
GO:0098531	transcription factor activity, direct ligand regulated sequence-specific DNA binding	1.79E-2	2.97
GO:0038023	signaling receptor activity	2.15E-2	2.65
GO:0003707	steroid hormone receptor activity	2.35E-2	2.7
GO:0004872	receptor activity	2.87E-2	2.65

Table S5. GO function term enrichment according to the sum measure $T(100; H_A, H_B)$ in the human all-tissues and brain network comparison.
