# Supplementary material for: Comparative analysis of weighted gene co-expression networks in human and mouse
Source: PLoS One. 2017 Nov 21;12(11):e0187611. doi: 10.1371/journal.pone.0187611 (PMC5697817; doi:10.1371/journal.pone.0187611)
Supplement: S6 Table — (PDF) [file pone.0187611.s010.pdf]

Table S6

GO Term	Description	FDR p-value	Enrichment
GO:0005125	cytokine activity	4.6E-9	3.19
GO:0001653	peptide receptor activity	1.54E-6	3.21
GO:0038023	signaling receptor activity	1.63E-6	1.69
GO:0008528	G-protein coupled peptide receptor activity	1.89E-6	3.18
GO:0004930	G-protein coupled receptor activity	3.33E-6	2.16
GO:0004888	transmembrane signaling receptor activity	1.59E-5	1.68
GO:0004872	receptor activity	2.02E-5	1.56
GO:0070405	ammonium ion binding	1.86E-4	3.69
GO:0030594	neurotransmitter receptor activity	2.53E-4	4.4
GO:0005126	cytokine receptor binding	5.34E-4	2.13
GO:0004871	signal transducer activity	5.83E-4	1.46
GO:0015464	acetylcholine receptor activity	1.09E-3	5.24
GO:0000981	RNA polymerase II transcription factor activity, sequence-specific DNA binding	1.98E-3	1.64
GO:0005230	extracellular ligand-gated ion channel activity	2.08E-3	2.99
GO:0060089	molecular transducer activity	2.53E-3	1.38
GO:0050997	quaternary ammonium group binding	2.41E-3	3.83
GO:0022834	ligand-gated channel activity	2.34E-3	2.45
GO:0015276	ligand-gated ion channel activity	2.21E-3	2.45
GO:0001071	nucleic acid binding transcription factor activity	2.37E-3	1.49
GO:0003700	transcription factor activity, sequence-specific DNA binding	2.25E-3	1.49
GO:0004977	melanocortin receptor activity	2.44E-3	9.43
GO:0004889	acetylcholine-activated cation-selective channel activity	4.56E-3	5.39
GO:0042166	acetylcholine binding	8.48E-3	5.03
GO:0022857	transmembrane transporter activity	9.27E-3	1.47
GO:0015075	ion transmembrane transporter activity	1.28E-2	1.5
GO:0004252	serine-type endopeptidase activity	1.35E-2	2.42
GO:0008083	growth factor activity	1.36E-2	2.14
GO:0022891	substrate-specific transmembrane transporter activity	1.8E-2	1.46
GO:0043565	sequence-specific DNA binding	2.29E-2	1.43
GO:0022803	passive transmembrane transporter activity	2.27E-2	1.66
GO:0015267	channel activity	2.2E-2	1.66
GO:0022838	substrate-specific channel activity	2.52E-2	1.68
GO:0003705	transcription factor activity, RNA polymerase II distal enhancer sequence-specific binding	2.66E-2	2.33
GO:0046933	proton-transporting ATP synthase activity, rotational mechanism	2.64E-2	6.74
GO:0005212	structural constituent of eye lens	2.83E-2	4.19
GO:0008324	cation transmembrane transporter activity	2.77E-2	1.54

GO:0005231	excitatory extracellular ligand-gated ion channel activity	3.62E-2	2.85

Table S6. GO function term enrichment according to the sum measure $T(1000; H_A, M_A)$ in the human and mouse all-tissues network comparison.
