# Supplementary material for: Comparative analysis of weighted gene co-expression networks in human and mouse
Source: PLoS One. 2017 Nov 21;12(11):e0187611. doi: 10.1371/journal.pone.0187611 (PMC5697817; doi:10.1371/journal.pone.0187611)
Supplement: S7 Table — (PDF) [file pone.0187611.s011.pdf]

Table S7

GO Term	Description	FDR p-value	Enrichment
GO:0046873	metal ion transmembrane transporter activity	1.48E-5	1.72
GO:0005249	voltage-gated potassium channel activity	1.03E-4	2.62
GO:0022836	gated channel activity	2.03E-4	1.73
GO:0005267	potassium channel activity	2.2E-4	2.28
GO:0015079	potassium ion transmembrane transporter activity	2.33E-4	2.12
GO:0015077	monovalent inorganic cation transmembrane transporter activity	2.24E-4	1.67
GO:0022890	inorganic cation transmembrane transporter activity	2.86E-4	1.54
GO:0022891	substrate-specific transmembrane transporter activity	2.95E-4	1.4
GO:0015075	ion transmembrane transporter activity	5.45E-4	1.4
GO:0022843	voltage-gated cation channel activity	5.05E-4	2.04
GO:0005216	ion channel activity	4.85E-4	1.59
GO:0022857	transmembrane transporter activity	6.26E-4	1.36
GO:0022832	voltage-gated channel activity	6.12E-4	1.91
GO:0005244	voltage-gated ion channel activity	5.69E-4	1.91
GO:0008324	cation transmembrane transporter activity	5.49E-4	1.46
GO:0022838	substrate-specific channel activity	1.01E-3	1.55
GO:0005198	structural molecule activity	1.12E-3	1.44
GO:0022803	passive transmembrane transporter activity	1.08E-3	1.52
GO:0015267	channel activity	1.02E-3	1.52
GO:0022892	substrate-specific transporter activity	1.37E-3	1.32
GO:0005215	transporter activity	1.63E-3	1.29
GO:0005261	cation channel activity	3.21E-3	1.62
GO:0008066	glutamate receptor activity	1.07E-2	2.91

Table S7. GO function term enrichment according to the human-centric difference measure $U(2000; H_A, M_A)$ in the human and mouse all-tissues network comparison.
