# Supplementary material for: Comparative analysis of weighted gene co-expression networks in human and mouse
Source: PLoS One. 2017 Nov 21;12(11):e0187611. doi: 10.1371/journal.pone.0187611 (PMC5697817; doi:10.1371/journal.pone.0187611)
Supplement: S8 Table — (PDF) [file pone.0187611.s012.pdf]

Table S8

GO Term	Description	FDR p-value	Enrichment
GO:0006259	DNA metabolic process	1.93E-9	1.67
GO:0006310	DNA recombination	6.46E-9	2.17
GO:0006281	DNA repair	1.15E-7	1.77
GO:0000278	mitotic cell cycle	3.32E-7	1.71
GO:0007049	cell cycle	3.05E-7	1.59
GO:0022402	cell cycle process	7.5E-7	1.44
GO:0002376	immune system process	8.29E-7	1.32
GO:0006302	double-strand break repair	4.04E-6	2.1
GO:0051276	chromosome organization	6.62E-6	1.79
GO:0000724	double-strand break repair via homologous recombination	6.33E-6	2.33
GO:0000725	recombinational repair	5.75E-6	2.33
GO:1903047	mitotic cell cycle process	1.29E-4	1.45
GO:0006260	DNA replication	2.82E-4	1.95
GO:0090304	nucleic acid metabolic process	2.86E-4	1.19
GO:0006955	immune response	3.56E-4	1.33
GO:0006974	cellular response to DNA damage stimulus	6.08E-4	1.43
GO:0006312	mitotic recombination	8.01E-4	2.85
GO:0044260	cellular macromolecule metabolic process	8E-4	1.11
GO:0007159	leukocyte cell-cell adhesion	9.22E-4	1.7
GO:0046649	lymphocyte activation	9.74E-4	1.61
GO:0006270	DNA replication initiation	9.34E-4	3.08
GO:0070486	leukocyte aggregation	1.07E-3	1.75
GO:0050776	regulation of immune response	1.07E-3	1.34
GO:0000722	telomere maintenance via recombination	1.77E-3	2.96
GO:0034109	homotypic cell-cell adhesion	1.91E-3	1.65
GO:0000083	regulation of transcription involved in G1/S transition of mitotic cell cycle	1.96E-3	3.29
GO:0006139	nucleobase-containing compound metabolic process	1.98E-3	1.15
GO:0032200	telomere organization	1.94E-3	2.21
GO:0022616	DNA strand elongation	2.02E-3	2.6
GO:1902589	single-organism organelle organization	2.03E-3	1.23
GO:0045003	double-strand break repair via synthesis-dependent strand annealing	1.97E-3	3.01
GO:0071593	lymphocyte aggregation	2.13E-3	1.73
GO:0002682	regulation of immune system process	2.29E-3	1.25
GO:0000723	telomere maintenance	2.44E-3	2.2
GO:0070489	T cell aggregation	2.76E-3	1.72
GO:0042110	T cell activation	2.68E-3	1.72
GO:0045321	leukocyte activation	2.82E-3	1.5
GO:0002250	adaptive immune response	3.17E-3	1.88
GO:0043170	macromolecule metabolic process	3.85E-3	1.09
GO:0002684	positive regulation of immune system process	6.41E-3	1.31
GO:0000075	cell cycle checkpoint	8.63E-3	1.81
GO:0006271	DNA strand elongation involved in DNA replication	8.63E-3	2.57

GO:0071897	DNA biosynthetic process	9.57E-3	2.12
GO:0031570	DNA integrity checkpoint	9.78E-3	2.15
GO:0006725	cellular aromatic compound metabolic process	9.66E-3	1.13
GO:0006325	chromatin organization	1E-2	1.39
GO:0042113	B cell activation	1.05E-2	1.84
GO:0006996	organelle organization	1.08E-2	1.17
GO:0046483	heterocycle metabolic process	1.24E-2	1.13
GO:0002504	antigen processing and presentation of peptide or polysaccharide antigen via MHC class II	1.25E-2	2.11
GO:0002495	antigen processing and presentation of peptide antigen via MHC class II	1.22E-2	2.11
GO:0019886	antigen processing and presentation of exogenous peptide antigen via MHC class II	1.2E-2	2.11
GO:0050865	regulation of cell activation	1.2E-2	1.42
GO:0000726	non-recombinational repair	1.28E-2	2.35
GO:0010389	regulation of G2/M transition of mitotic cell cycle	1.26E-2	2.35
GO:0010833	telomere maintenance via telomere lengthening	1.24E-2	2.35
GO:0006303	double-strand break repair via nonhomologous end joining	1.22E-2	2.35
GO:0006950	response to stress	1.36E-2	1.14
GO:0048285	organelle fission	1.38E-2	1.54
GO:0032201	telomere maintenance via semi-conservative replication	1.8E-2	2.75
GO:0001775	cell activation	2.39E-2	1.33
GO:0002699	positive regulation of immune effector process	2.37E-2	1.7
GO:0000280	nuclear division	2.37E-2	1.54
GO:0016568	chromatin modification	2.42E-2	1.39
GO:1902749	regulation of cell cycle G2/M phase transition	2.56E-2	2.24
GO:0002703	regulation of leukocyte mediated immunity	2.58E-2	1.72
GO:0050867	positive regulation of cell activation	2.64E-2	1.51
GO:1903046	meiotic cell cycle process	3.2E-2	1.79
GO:0016337	single organismal cell-cell adhesion	3.2E-2	1.4
GO:0098602	single organism cell adhesion	3.17E-2	1.39
GO:0034644	cellular response to UV	3.23E-2	2.11
GO:0033554	cellular response to stress	3.27E-2	1.21
GO:0071103	DNA conformation change	4.03E-2	1.86
GO:0051249	regulation of lymphocyte activation	4.05E-2	1.43
GO:0043412	macromolecule modification	4.21E-2	1.14
GO:0002697	regulation of immune effector process	4.63E-2	1.41
GO:0002696	positive regulation of leukocyte activation	4.62E-2	1.49
GO:0051251	positive regulation of lymphocyte activation	4.64E-2	1.52

GO:0006297	nucleotide-excision repair, DNA gap filling	4.65E-2	2.88
GO:0072676	lymphocyte migration	4.87E-2	2.35

Table S8. GO process term enrichment according to the mouse-centric difference measure $U(2000; M_A, H_A)$ in the human and mouse all-tissues network comparison.
